# Altered brain dynamics index levels of arousal in complete locked-in syndrome

**DOI:** 10.1038/s42003-023-05109-1

**Published:** 2023-07-20

**Authors:** Federico Zilio, Javier Gomez-Pilar, Ujwal Chaudhary, Stuart Fogel, Tatiana Fomina, Matthis Synofzik, Ludger Schöls, Shumei Cao, Jun Zhang, Zirui Huang, Niels Birbaumer, Georg Northoff

**Affiliations:** 1 Department of Philosophy, Sociology, Education and Applied Psychology, University of Padova, Padua, Italy; 2 Biomedical Engineering Group, University of Valladolid, Valladolid, Spain; 3 Centro de Investigación Biomédica en Red en Bioingeniería, Biomateriales y Nanomedicina (CIBER-BBN), Valladolid, Spain; 4 BrainPortal Technologies GmbH, Mannheim, Germany; 5 ALS Voice gGmbH, Mössingen, Germany; 6 School of Psychology, University of Ottawa, Ottawa, Canada; 7 Institute of Mental Health Research, University of Ottawa, Ottawa, Canada; 8 Department for Empirical Inference, Max Planck Institute for Intelligent Systems, Tübingen, Germany; 9 Department of Neurodegenerative Diseases and Hertie-Institute for Clinical Brain Research, University of Tübingen, Tübingen, Germany; 10 German Center for Neurodegenerative Diseases (DZNE), Tübingen, Germany; 11 Department of Anesthesiology, Fudan University Shanghai Cancer Center, Shanghai, China; 12 Center for Consciousness Science, Department of Anesthesiology, University of Michigan Medical School, Ann Arbor, MI USA; 13 Institute of Medical Psychology and Behavioral Neurobiology, University of Tübingen, Tübingen, Germany

**Keywords:** Consciousness, Amyotrophic lateral sclerosis

## Abstract

Complete locked-in syndrome (CLIS) resulting from late-stage amyotrophic lateral sclerosis (ALS) is characterised by loss of motor function and eye movements. The absence of behavioural indicators of consciousness makes the search for neuronal correlates as possible biomarkers clinically and ethically urgent. EEG-based measures of brain dynamics such as power-law exponent (PLE) and Lempel-Ziv complexity (LZC) have been shown to have explanatory power for consciousness and may provide such neuronal indices for patients with CLIS. Here, we validated PLE and LZC (calculated in a dynamic way) as benchmarks of a wide range of arousal states across different reference states of consciousness (e.g., awake, sleep stages, ketamine, sevoflurane). We show a tendency toward high PLE and low LZC, with high intra-subject fluctuations and inter-subject variability in a cohort of CLIS patients with values graded along different arousal states as in our reference data sets. In conclusion, changes in brain dynamics indicate altered arousal in CLIS. Specifically, PLE and LZC are potentially relevant biomarkers to identify or diagnose the arousal level in CLIS and to determine the optimal time point for treatment, including communication attempts.

## Abstract (web summary)

EEG-based dynamics associated with different arousal states are used to identify indices associated with complete locked-in syndrome, which could aid treatments and communication aids

## Introduction

Locked-in syndrome (LIS) is a pathological condition in which patients cannot move due to motor paralysis while their consciousness is preserved^1,2^. LIS is more frequently caused by local brain injury^3^, but can also be observed in the late stage of amyotrophic lateral sclerosis (ALS)^2,4,5^. As ALS progresses, patients gradually lose the ability to move, talk, swallow, and breathe autonomously. Communication is possible, but, mostly through eye movements; alone, or combined with a brain-computer interface (BCI), which can provide the ability to exchange thoughts and feelings with others^6^. However, a portion of patients with ALS eventually converts to what is called complete locked-in syndrome (CLIS), characterised by the loss of reliable eye movements and other motor functions^2^. In this state, communication through eye movements is no longer reliable. While some eye movements may persist, these individuals are no longer functional for communication, and BCI communication becomes impossible^7–16^ (note: except for a recent case where communication was established through invasive BCI^9^). It is still unclear why patients with CLIS-ALS are generally unable to use BCI communication. It has been hypothesized that a progressive reduction or even extinction of goal-directed thinking in complete paralysis^11^, namely, the complete lack of motor control and feedback could be responsible for the cessation of voluntary cognitive activity, goal-directed thinking, and mental imagery^11,15,17,18^. However, we currently lack the means to probe the state of consciousness of these patients and similar, or related conditions^9^. Thus, the search for neuronal markers that index either states, or levels of consciousness is rather urgent in CLIS patients. That is the main goal of this study, which, in addition to providing scientific evidence, has major ethical and clinical implications (see discussion).

One hallmark feature of CLIS is the difficulty, or even the impossibility, of communication of any kind (either with, or without brain-computer interface—BCI). It is possible that this may be in part due to fluctuations in cognitive abilities, attention, and alertness over the course of the day and/or a worsening of such abilities over time^6,10,11,15,18–23^. Some cases of CLIS are clinically characterised by changes in circadian rhythm and sleep patterns inferred from the fragmentation of slow-wave sleep and the absence of sleep spindles^24,25^, and by alterations of alpha oscillations, reactivity, and peak frequency;^26,27^ which might suggest related alterations in arousal and attention during BCI communication attempts^20,26,28,29^. For example, it has been shown that, in a patient with CLIS-ALS, the alpha rhythm (a marker of relaxed wakefulness) fluctuated substantially over a 10-hour period^20^. In addition, reduced modulation of alpha and gamma power in the medial prefrontal cortex in CLIS patients compared to healthy controls in reaction to self-referential stimuli (‘self‘ vs. ‘friend’ and ‘celebrity’), suggesting alterations in self-referential thinking processes^19^. Finally, a patient with late-stage ALS found that, after the transition from LIS to CLIS, P300 responses from auditory stimuli could no longer be detected or conclusively reveal information processing^30^. Together, these findings do not constitute conclusive evidence for impairment of consciousness or cognitive abilities per se but suggest that patients with CLIS-ALS may be subject to alterations in consciousness or experience ‘windows’^31^ of consciousness.

Do these alterations in electrophysiological states reflect alterations in the patient’s state of consciousness? It is unclear which dimension of consciousness is potentially associated with these alterations, whether it be arousal (i.e., the overall state of alertness or wakefulness) or awareness (i.e., contents of consciousness), or even cognitive access^32–34^. In that case, intra-subject fluctuations in neural activity may be useful to index changes in arousal. Specifically, intra-subject fluctuations over time can be measured using dynamic analyses^35–37^. Specifically, a sliding window approach can be applied to our measures of brain dynamics, such as power-law exponent (a metric of the broadband non-periodic, arrhythmic quality of the EEG; PLE)^35–37^ and information-based Lempel-Ziv Complexity (a metric of how regular/repeatable, or diverse the EEG signal is over time; LZC). These measurements are altered in anaesthesia, disorders of consciousness, and sleep^38–43^. Given our hypothesis in regards to altered arousal in CLIS, and given the relationship of static PLE/LZC to the level of arousal/wakefulness, we predict that the dynamic fluctuations of PLE/LZC will serve as an index of the alterations in the level of arousal/wakefulness in CLIS.

The goal of this study is to investigate the brain dynamics using EEG recordings, namely scale-free arrhythmic activity (PLE) and signal complexity (LZC), to probe whether they can serve as indices of the level of arousal (or wakefulness) in CLIS, at both the group and individual levels. LZC is one of the most consistent and reproducible measures in the context of consciousness, and PLE variations are robustly reflected in sleep, anaesthesia, and disorders of consciousness. LZC and PLE measure complementary characteristics, and the robustness and consistency across consciousness studies make them ideal candidates for our study.

For that, we used a dataset of CLIS patients and compared them to healthy control groups across a spectrum of different conscious states, including: anaesthesia (ketamine, sevoflurane), sleep (N1-2-3, REM), and LIS/non-LIS patients affected by ALS. We hypothesised that: (1) changes in both PLE would be higher than controls and LZC would be lower than controls in patients with CLIS, and (2) a greater degree of intra- and inter-subject variability (over time) in PLE/LZC in patients with CLIS would indicate corresponding alterations in arousal. To test these main hypotheses, three specific research objectives are necessary based on specific secondary hypotheses:

1. The first specific objective consisted of measuring PLE and LZC from the EEG as benchmarks of normal variations across states of consciousness. To this end, we investigated the mean and the coefficient of variation (CV) of PLE and LZC in different states such as sleep (from wakefulness to N1, N2, N3, and REM) and anaesthesia (sevoflurane, ketamine). We hypothesised that PLE increases parametrically in states associated with an assumed decrease or absence of arousal/wakefulness, while we hypothesised that LZC decreases in states. In addition to characterising PLE and LZC using mean and the CV, we further probed the utility of these indices employing receiver operating characteristic (ROC) curve as a measure of their classification robustness in distinguishing conscious *vs*. alterations of conscious states in these conditions. This objective will demonstrate that PLE and LZC can be useful indices of the level of arousal across different states of consciousness.
2. The second objective was to apply PLE and LZC to a group of participants with CLIS (*n* = 12) to investigate their level of arousal; in particular, four of these patients received a series of repeated EEG recordings, which also allowed us to investigate changes in PLE and LZC between sessions in the same participant. We hypothesised that patients with CLIS would show altered PLE and LZC compared to controls, with varying degrees of mean and CV at both the group and individual levels. This would distinguish them from both LIS and non-LIS patients, as well as healthy participants, suggesting a potentially degraded state of wakefulness in patients with CLIS over a prolonged period of time. This objective will demonstrate whether and how the level of arousal is altered in patients with CLIS.
3. The third objective consisted of measuring the relationship between PLE and LZC, i.e., between arrhythmicity and signal complexity, respectively^42^. Given our previous findings^44,45^ and others^46–48^ in healthy awake participants, we predicted that PLE and LZC will negatively correlate in a non-linear way. This will be validated by using both empirical data from the analysed datasets, and also a simulation model using synthetic signals generated by varying the noise power decay factor with frequency. The imbalance between slow and fast frequencies with a progressive shift towards slower frequencies (PLE) can be accompanied by loss of information complexity (LZC) during the alteration of arousal in CLIS and analogous states (from reduced arousal in N1, ketamine, REM to abolished arousal in N2, N3, sevoflurane). This objective will demonstrate whether changes in arousal level with their power spectrum changes are related to reduced information processing in CLIS patients.

**Fig. 1:**
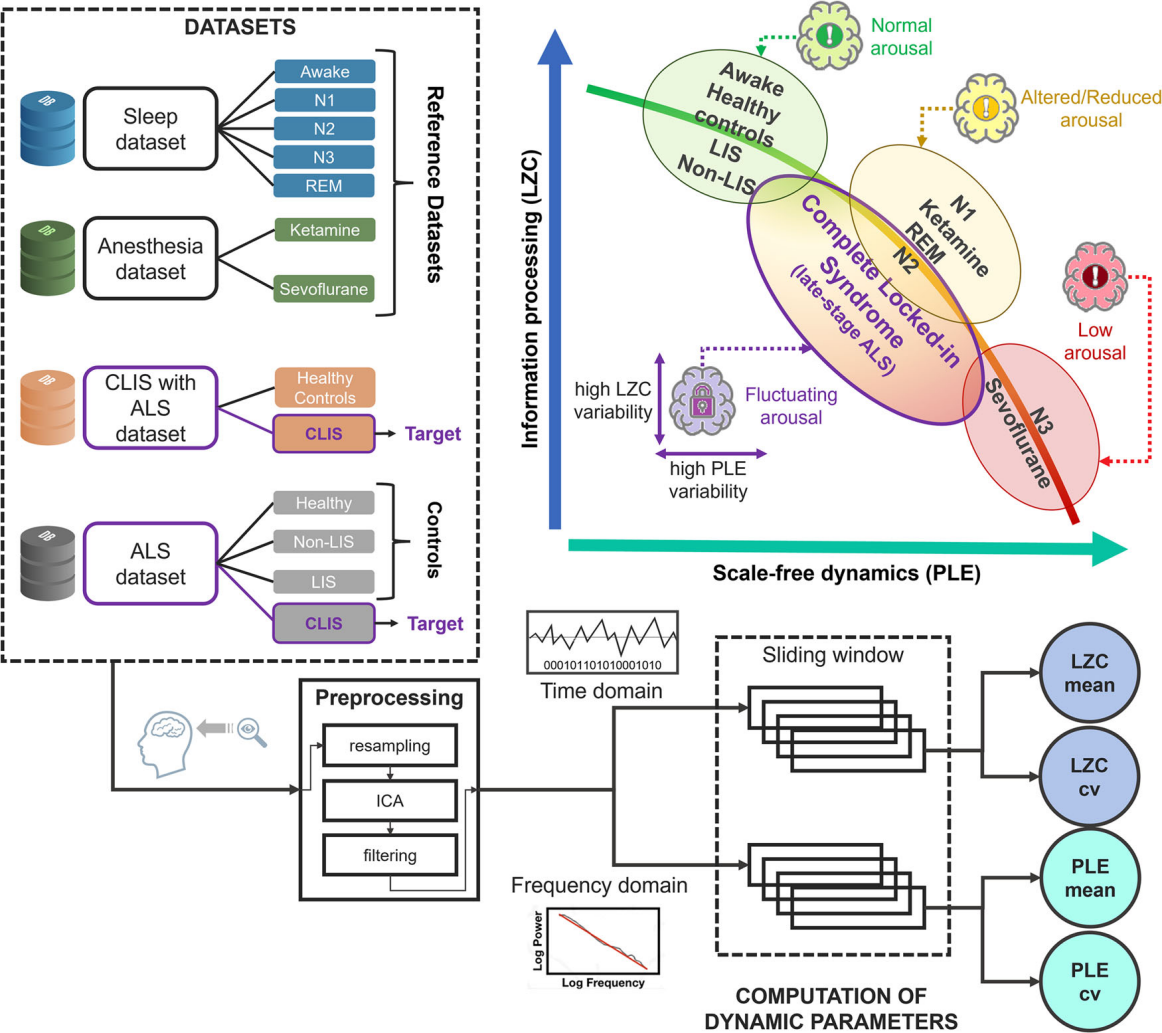
Schematic of the rationale and methodology of the study. Through the analysis of four datasets characterised by normal and altered levels of arousal, the non-linear relationship between PLE and LZC (mean and coefficient of variation taken across sliding windows) is identified. Once the association between brain dynamics, information processing, and wakefulness is validated, the results from the various states (healthy controls, sleep stages, and anaesthesia) are compared with the altered values of PLE and LZC and the high degree of inter-subject and intra-subject variability among participants with CLIS.

Our multigroup EEG study shows the utility of PLE and LZC to serve as a benchmark, and thus, index the level of alertness/wakefulness in CLIS. This is supported by both empirical data and a simulation model. We demonstrate that a shift towards slower frequencies (high PLE) is related to a decrease in processing information complexity (low LZC), which, at least in part, may reflect a reduction in arousal in CLIS. This may consequently compromise communication. Taken together, we present empirical evidence for reduced and highly unstable and fluctuating brain dynamics (PLE) and signal complexity (LZC) in CLIS reflecting intra-subject fluctuations in their level of arousal/state of alertness (Fig. 1).

## Results

### Sleep

**Fig. 2:**
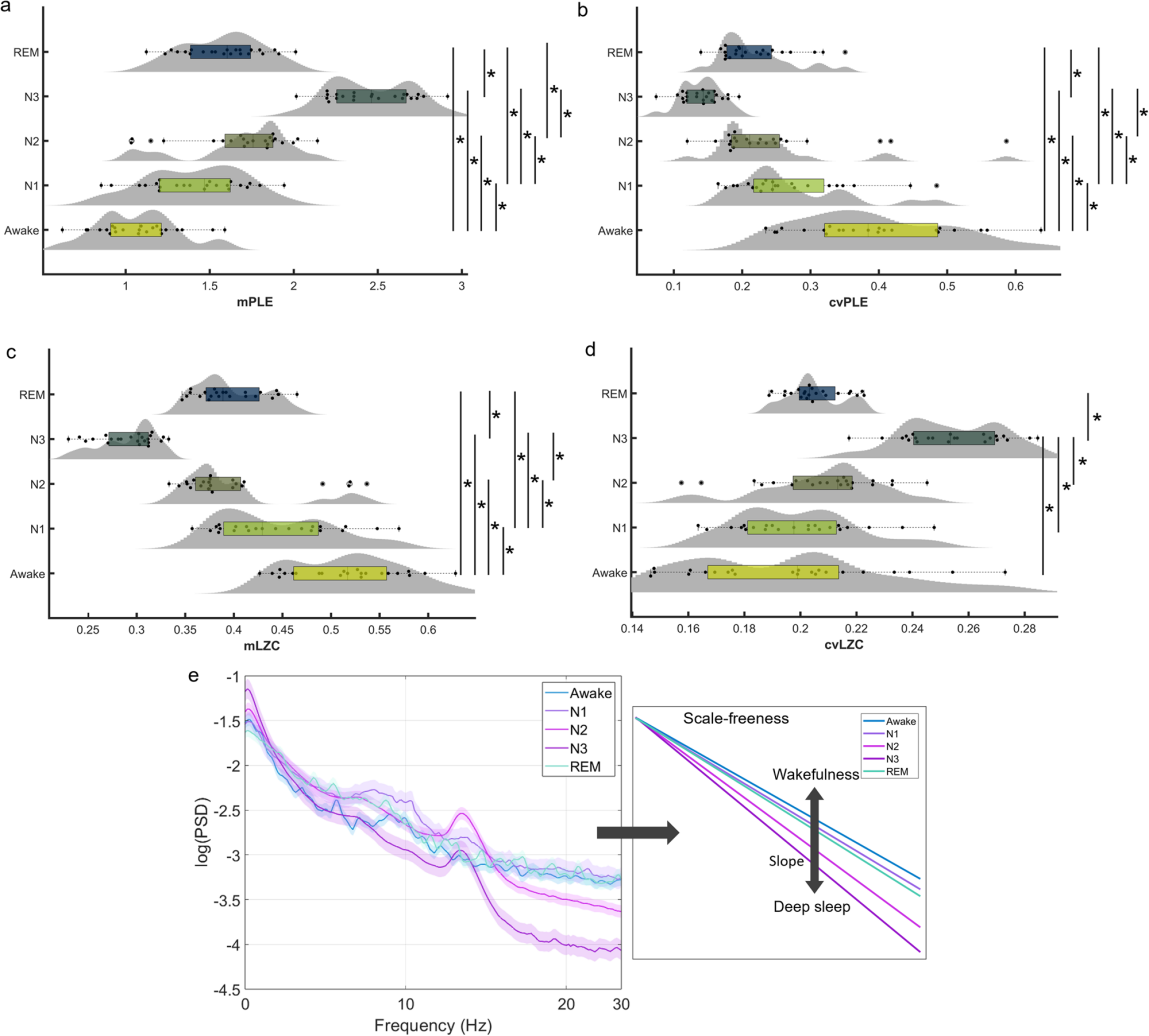
Evaluation of PLE and LZC in the sleep dataset. The mean PLE (**a**) and LZC (**c**) distributions and their respective coefficients of variation (**b**, **d**) are depicted using violin plots and box plots for each of the sleep stages. A significant increase for mPLE (with a decrease for cvPLE) and a significant decrease for mLZC (with an increase for cvLZC) are observed for deeper sleep stages. Mean PSD with standard error mean (**e**) is represented for awake and all sleep stages, showing that the scale-free structure of the power spectrum changes in N1, N2, N3, and REM, as the relationship in power between slow and fast frequencies is altered, with a shift towards lower frequencies (except for REM, which is most similar to the light sleep stage N1 and the waking state). Source data are provided in Supplementary Data 1.

Our results showed an increase in the mean PLE (mPLE) and coefficient of variation of LZC (cvLZC), and a decrease in mean LZC (mLZC) and coefficient of variation of PLE (cvPLE) as the sleep stages deepened (Awake, N1, N2, N3). In almost all cases, REM sleep values fell between the waking state and deeper stages of sleep (Fig. 2a–d). Similarly, PSD showed a progressive increase in power at slow frequencies (1–8 Hz) and decreased power at higher frequencies (10–40 Hz) from wakefulness to deep sleep, except for the REM phase, which showed a power spectrum most similar to N1 and wakefulness. This is consistent with the neurophysiology and cognitive state of REM sleep, which is paradoxically wake-like^49^ and is a sleep state typically (but not the only one) characterised by vivid dream content^50^. This is also reflected by the different degrees of slope of the scale-free structure of the PSD (Fig. 2e), where the steepest slope is in N3, the flattest in the waking state, and again, the slope for REM sleep is slightly below N1. Repeated measures test showed statistically significant differences between all states analysed here (*n* = 23, *p* < 0.001 and *p* < 0.05, see Table 1), with a few exceptions where *p* values > 0.05 were found for the comparisons between N2 and REM (mLZC: N2-REM; cvPLE: N2-REM; cvLZC: Awake-N1-N2-REM, N1-N2-REM, N2-REM). The coefficient of variation of the LZC was found to be the measurement with the fewest significant differences. However, the ROCs showed significantly higher performance than the chance level in all measurements (AUC: mPLE = 0.92; cvPLE = 0.91; mLZC = 0.92; cvLZC = 0.68), supporting the robustness of the significant differences shown in the box plots (see Supplementary Fig. 1). Finally, in all measurements, progressive global changes were observed in the topographic maps, which is supported by generally widespread statistical differences (repeated measures test) after FDR correction (Benjamini-Yekutieli) found in most comparisons between sleep states (see Supplementary Fig. 1). Thus, taken together, PLE and LZC are robust metrics of the temporal signatures of the EEG that can discriminate well between the various sleep-wake states in a predictable manner.

**Table 1:** Main results of all the datasets analysed: mean, standard deviation, and statistical comparisons.

Sleep					
	Mean and standard deviation				
	Awake	N1	N2	N3	REM
mPLE	1.07 ± 0.24	1.41 ± 0.29	1.69 ± 0.31	2.45 ± 0.24	1.57 ± 0.24
mLZC	0.52 ± 0.06	0.44 ± 0.06	0.40 ± 0.06	0.29 ± 0.03	0.39 ± 0.03
cvPLE	0.39 ± 0.11	0.27 ± 0.08	0.24 ± 0.10	0.03 ± 0.03	0.05 ± 0.05
cvLZC	0.19 ± 0.03	0.20 ± 0.02	0.20 ± 0.02	0.25 ± 0.02	0.21 ± 0.01

	*p* values									
	Awake vs N1	Awake vs. N2	Awake vs. N3	Awake vs. REM	N1 vs. N2	N1 vs. N3	N1 vs. REM	N2 vs. N3	N2 vs. REM	N3 vs. REM
mPLE	<0.001	<0.001	<0.001	<0.001	<0.001	<0.001	<0.5	<0.001	<0.05	<0.001
mLZC	<0.001	<0.001	<0.001	<0.001	<0.001	<0.001	<0.001	<0.001	NS	<0.001
cvPLE	<0.001	<0.001	<0.001	<0.001	<0.05	<0.001	<0.05	<0.001	NS	<0.001
cvLZC	NS	NS	<0.001	NS	NS	<0.001	NS	<0.001	NS	<0.001

	Anaesthesia			
	Mean and standard deviation			
	Pre-ketamine	Ketamine	Pre-sevoflurane	Sevoflurane
mPLE	1.16 ± 0.26	1.43 ± 0.27	1.12 ± 0.22	2.30 ± 0.40
mLZC	0.48 ± 0.07	0.42 ± 0.05	0.56 ± 0.06	0.25 ± 0.06
cvPLE	0.30 ± 0.07	0.23 ± 0.07	0.29 ± 0.05	0.14 ± 0.04
cvLZC	0.18 ± 0.04	0.20 ± 0.02	0.17 ± 0.02	0.23 ± 0.04

	*p* values			
	Pre-ketamine vs. ketamine		Pre-sevoflurane vs. sevoflurane	
mPLE	<0.05		<0.05	
mLZC	NS		<0.05	
cvPLE	NS		<0.05	
cvLZC	NS		<0.05	

	Complete locked-in syndrome	
	Mean and standard deviation	
	Healthy controls	CLIS
mPLE	0.97 ± 0.40	2.11 ± 0.65
mLZC	0.55 ± 0.08	0.34 ± 0.09
cvPLE	0.44 ± 0.27	0.16 ± 0.04
cvLZC	0.16 ± 0.04	0.22 ± 0.04

	*p* values	
	HC vs. CLIS	
mPLE	<0.001	
mLZC	<0.001	
cvPLE	<0.001	
cvLZC	<0.05	

	3 CLIS patients–multisession		
	Mean and standard deviation		
	CLIS P#6	CLIS P#9	CLIS P#11
mPLE	2.07 ± 0.21	1.85 ± 0.17	1.17 ± 0.08
mLZC	0.34 ± 0.03	0.37 ± 0.03	0.50 ± 0.02
cvPLE	0.22 ± 0.06	0.20 ± 0.02	0.27 ± 0.03
cvLZC	0.29 ± 0.03	0.25 ± 0.02	0.18 ± 0.01

	*p* values		
	P#6 vs. P#9 vs. *P*#11		
mPLE	<0.001		
mLZC	<0.001		
cvPLE	<0.001		
cvLZC	<0.001		

	Amyotrophic lateral sclerosis			
	Mean and standard deviation			
	Healthy controls	non-LIS	LIS	CLIS
mPLE	0.86 ± 0.24	0.73 ± 0.29	0.86	1.97 ± 0.03
mLZC	0.56 ± 0.06	0.60 ± 0.08	0.62	0.36 ± 0.03
cvPLE	0.41 ± 0.12	0.47 ± 0.14	0.40	0.21 ± 0.01
cvLZC	0.15 ± 0.02	0.13 ± 0.02	0.16	0.28 ± 0.02

	*p* values	*z* scores		
	Healthy controls vs. nonLIS	LIS	CLIS 1	CLIS 2
mPLE	NS	<1.96	>1.96	>1.96
mLZC	NS	<1.96	>1.96	>1.96
cvPLE	NS	<1.96	<1.96	<1.96
cvLZC	<0.05	<1.96	>1.96	>1.96

	CLIS—non-LIS	
	Mean and standard deviation	
	CLIS 1	Non-LIS
mPLE	1.56 ± 0.43	0.64 ± 0.09
mLZC	0.47 ± 0.10	0.61 ± 0.02
cvPLE	0.37 ± 0.17	0.63 ± 0.16
cvLZC	0.28 ± 0.06	0.14 ± 0.01

	*p* values	
	CLIS 1 vs. nonLIS	
mPLE	<0.001	
mLZC	<0.001	
cvPLE	<0.001	
cvLZC	<0.001	

*NS* not significant.

### Anaesthesia

**Fig. 3:**
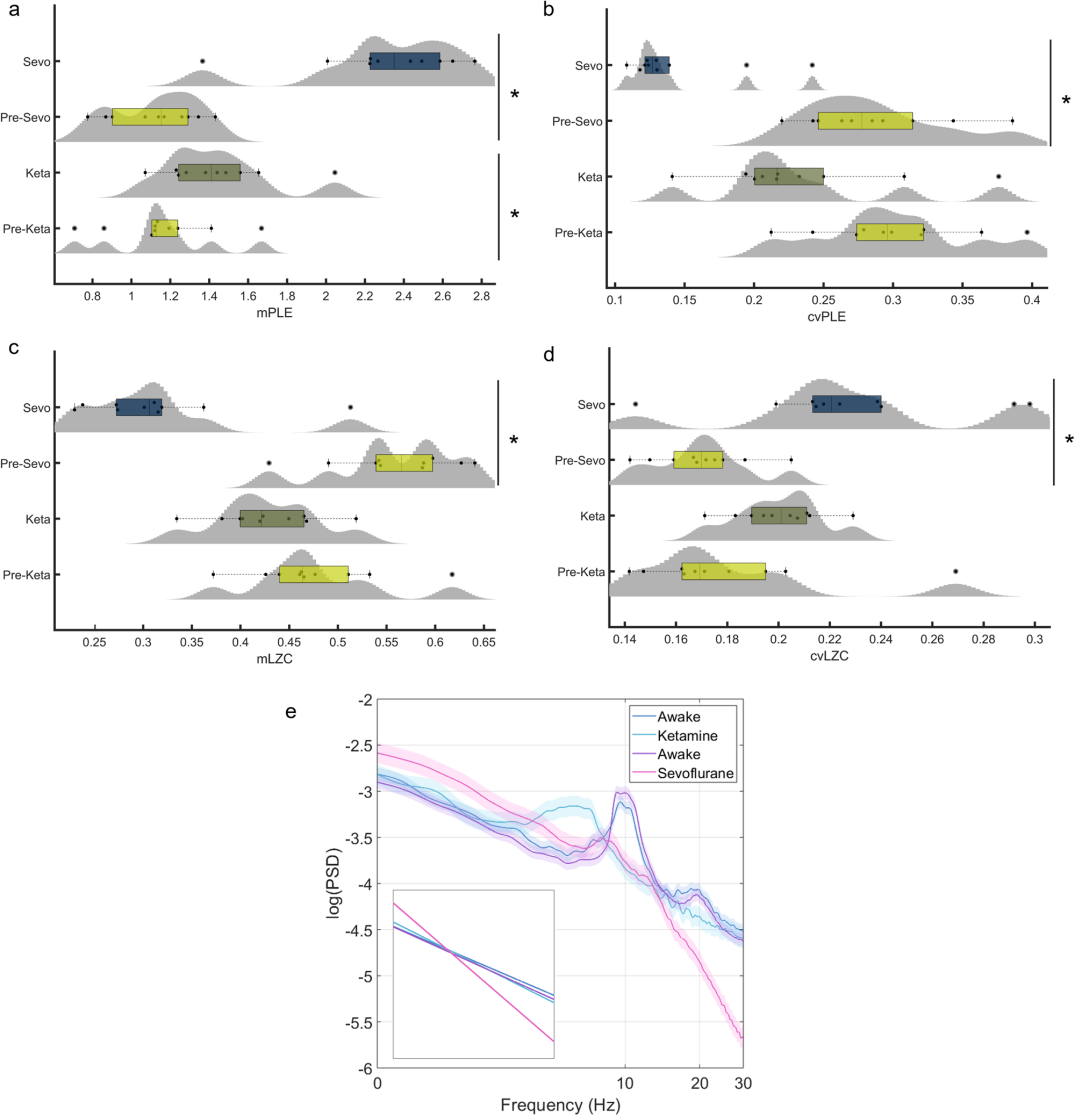
Evaluation of PLE and LZC in the anaesthesia dataset. The mean distributions of PLE (**a**) and LZC (**c**), and their respective coefficients of variation (**b**, **d**) are depicted using violin plots and box plots for each anaesthesia and pre-anaesthesia condition. A significant increase for mPLE (with a decrease for cvPLE) and a significant decrease for mLZC (with an increase for cvLZC) are observed for sevoflurane as compared to that observed in wakefulness through the measures test. A similar but less robust trend is shown for ketamine as compared to that for awake through a repeated measures test in mPLE (**a**). These results can be confirmed by visual inspection of the PSD with standard error mean (**e**). Source data are provided in Supplementary Data 1.

As predicted, participants who received sevoflurane showed a higher mPLE and, conversely, lower mLZC compared to the results shown by the same participants in the awake condition. Furthermore, cvPLE was lower during sevoflurane, while cvLZC was higher than in the awake condition. As expected, this is comparable to the results observed for N3 in the sleep dataset. The repeated measures test showed statistically significant differences between the awake and sevoflurane conditions (*n* = 10) in both mPLE (*p* < 0.05) and mLZC (*p* < 0.05) for the mean distribution of all electrodes, and in both cvPLE (*p* < 0.05) and cvLZC (*p* < 0.05) (Fig. 3a–d).

A similar trend was found for the ketamine condition, for mPLE, mLZC, cvPLE, and cvLZC. However, the increases in mPLE and the decrease in mLZC, as well as the decrease in cvPLE and the increase in cvLZC, were much less apparent as compared to the awake state than in the sevoflurane group. In this case, significant differences were found only for mPLE (repeated-measures test, *n* = 10, *p* < 0.05) (Fig. 3a). mLZC, cvPLE and cvLZC under ketamine conditions did not show significant statistical differences from the awake state (repeated-measures test, *p* > 0.05). However, ROC curves showed a significantly higher performance than chance in all measurements (AUC: mPLE = 0.79; cvPLE = 0.80; mLZC = 0.72; cvLZC = 0.80), highlighting still relevant robustness of the trend shown in the box plots (see Supplementary Fig. 2). These results are consistent with other studies showing that ketamine anaesthesia, despite behavioural unresponsiveness, preserves a dream-like state of consciousness, as reported by participants after awakening^33,42,51–53^.

The differences between the effects of sevoflurane and ketamine correspond well to the visual inspection of the PSD (Fig. 3e), whereby sevoflurane showed a generally steep decline of PSD compared to the awake state, with higher values at slow frequencies (1–8 Hz), a flattening of the alpha peak and a deeper slope at higher frequencies (20–40 Hz). By contrast, ketamine showed a slight flattening of PSD, with a slowdown and a shift of the alpha peak towards lower frequencies. Topographic maps for the differences between pre-anaesthesia and anaesthesia in mPLE, cvPLE, mLZC, and cvLZC showed global changes for sevoflurane and regional changes for ketamine. Statistical differences (repeated measures test) after FDR correction (Benjamini–Hochberg) can be seen in Supplementary Fig. 2. Thus, taken together, PLE and LZC are robust metrics of the temporal signatures of the EEG that can discriminate well between the wake state and anaesthesia in a predictable manner, most robustly for sevoflurane and less-so for ketamine.

### CLIS at the group and individual levels

**Fig. 4:**
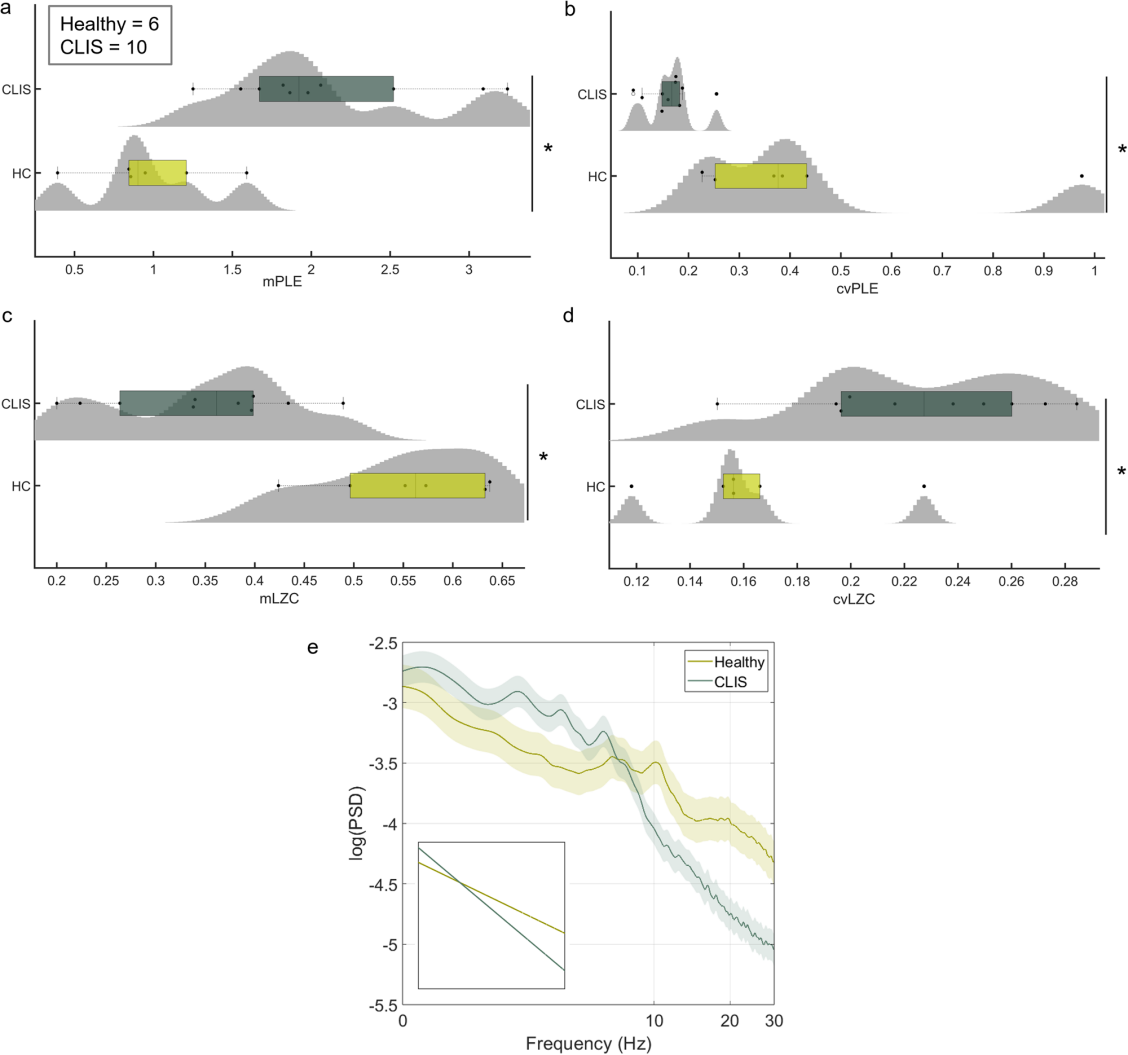
PLE and LZC assessment in the CLIS dataset. The mean distribution of PLE (**a**) and LZC (**c**) and their respective coefficients of variation (**b**, **d**) are depicted using violin plots and box plots for both the CLIS condition and healthy controls. A significant increase for mPLE (with a uniform decrease for cvPLE) and a significant decrease for mLZC (with an increase for cvLZC) are observed for CLIS patients compared to HC through non-repeated measures test. The PSD with standard error mean (**e**) of the 10 CLIS participants showed a steep overall decline compared to HC, with higher values in slow frequencies (1–8 Hz), a slowing of alpha peaks in the delta-theta range, and a power slope the higher frequencies (10–40 Hz). Source data are provided in Supplementary Data 1.

The ten participants with late-stage ALS and CLIS had a comparable pattern of results for sleep and anaesthesia; showing higher mPLE and, conversely, lower mLZC compared to healthy controls. Furthermore, in the CLIS participants, cvPLE was lower, while cvLZC was higher than in the healthy group. Non-repeated measures tests showed statistically significant differences between CLIS participants (*n* = 10) and healthy controls (*n* = 6) in both mPLE (*p* < 0.001) and mLZC (*p* < 0.001) for the grand average over the electrodes, and in both cvPLE (*p* < 0.001) and cvLZC (*p* < 0.05) (Fig. 4a–d) (for ROC curves, see Supplementary Fig. 3). In addition, the general pattern of differences for CLIS and healthy participants correspond well to the visual inspection of PSD (Fig. 4e), whereby CLIS patients showed a generally steep decline in PSD compared to healthy controls, with higher values at slow frequencies (1–8 Hz), a slowdown and shift in the alpha peak towards lower frequencies, and a negative slope at higher frequencies (10–40 Hz). Due to the use of different electrode locations, depending on the clinical requirements of each CLIS patients, it is not possible to perform an average of measures between participants for specific electrodes. Therefore, it is not possible to represent the usual topographic plots or perform a reliable statistical analysis for direct comparisons. Thus, a global analysis (average across electrodes) was performed. Taken together, these results suggest that PLE and LZC can discriminate well between the temporal variations in the EEG of patients with CLIS and healthy controls that may be clinically significant and have diagnostic and prognostic value.

### PLE and LZC alterations in three patients with ALS-CLIS

**Fig. 5:**
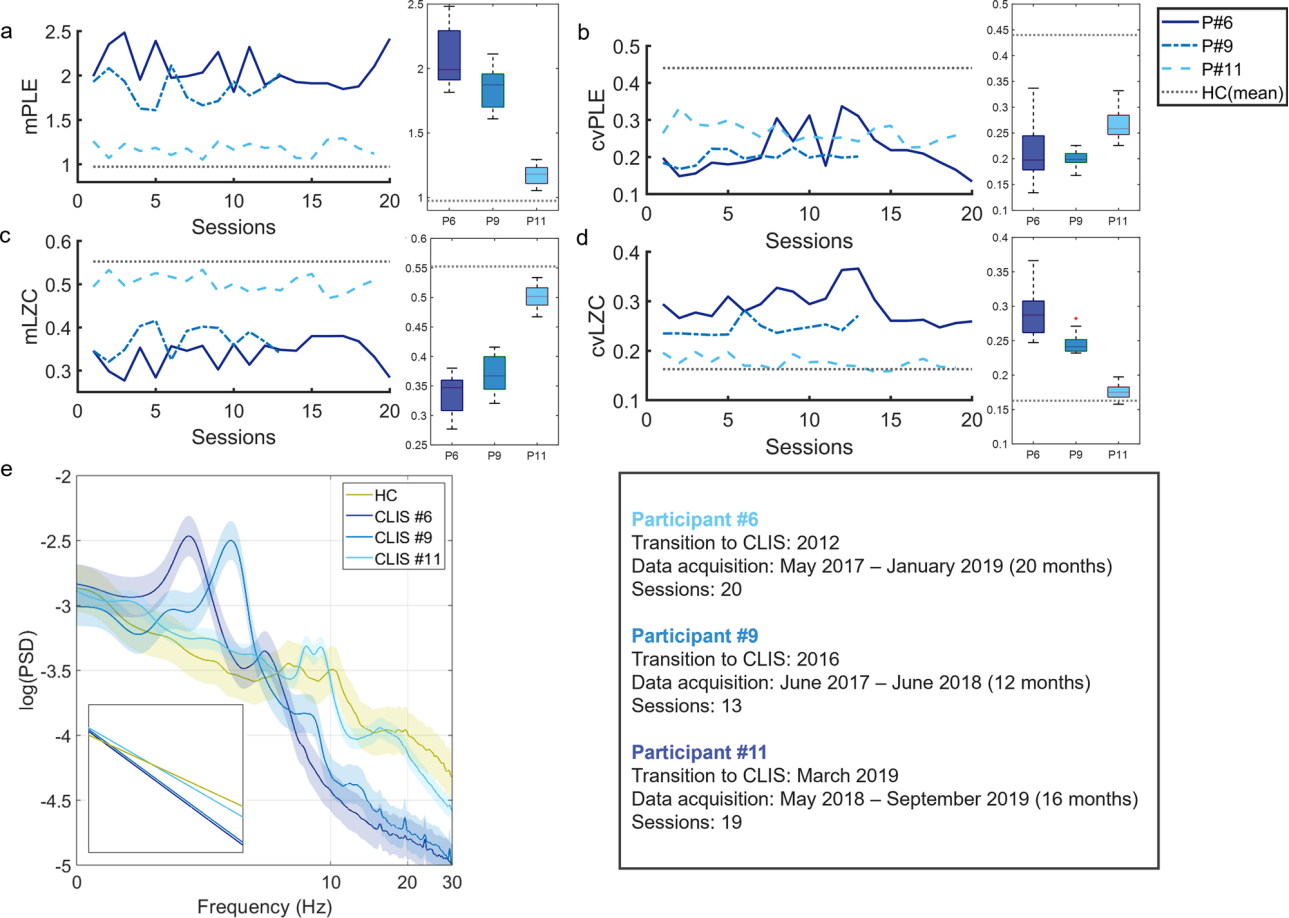
Longitudinal evaluation of PLE and LZC in three CLIS participants from the CLIS dataset (fluctuations between different sessions). The mean PLE (**a**) and LZC (**c**) distributions and their respective coefficients of variation (**b**, **d**) are represented using box plots for the three CLIS participants and also for the mean of the healthy controls group (n.b., multisession records in controls are missing). A progressive increase for mPLE (with a decrease for cvPLE) and a progressive decrease for mLZC (with an increase for cvLZC) are observed for the three CLIS participants compared to the average of the HC. PSD with standard error mean (**e**) showed a power spectrum close to the mean of HC for participant #11 (transition to CLIS occurred during the EEG recording period), while PSD of participants #9 (1 year in CLIS) and #11 (5 years in CLIS) showed an overall steep decline compared to HC, with higher values in slow frequencies (1–8 Hz), a slowing of alpha peaks in the delta-theta range and a loss of power in higher frequencies (10–40 Hz). Source data are provided in Supplementary Data 1.

**Fig. 6:**
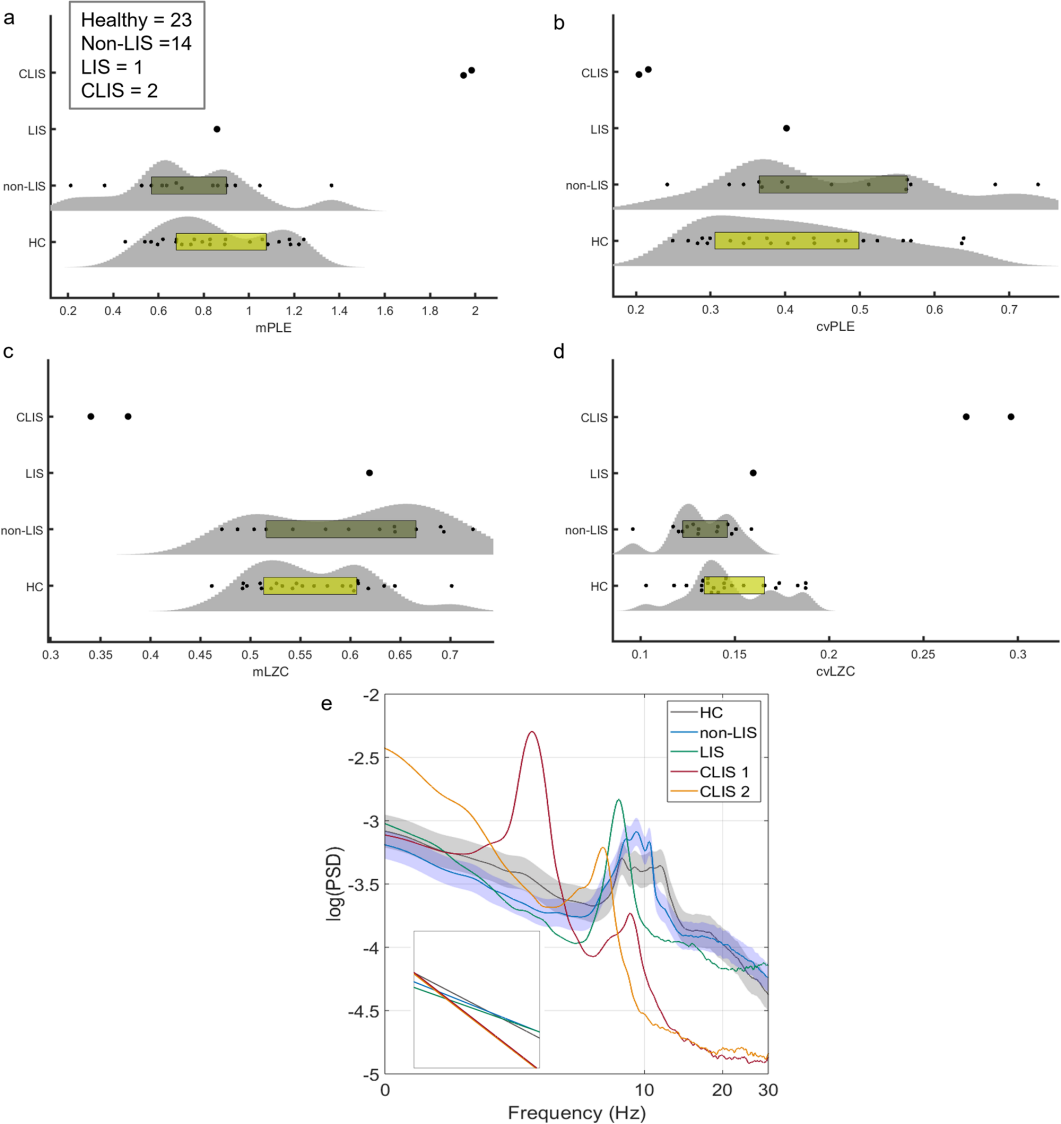
Evaluation of PLE and LZC in the ALS dataset. The mean distribution of PLE (**a**) and LZC (**c**) and their respective coefficients of variation (**b**, **d**) are represented using violin plots and box plots for the two CLIS patients, the LIS participant, the 14 non-LIS ALS participants, and the healthy controls. A significant increase for mPLE (with a decrease for cvPLE) and a significant decrease for mLZC (with an increase for cvLZC) are observed only for the CLIS participants compared to the other groups through *z* score normalisation. Instead, the results observed in the HC, non-LIS, and LIS groups were not significant by non-repeated measures test (HC and non-LIS) and *z* score normalisation (the single LIS patient), except for a significant decrease for cvLZC in participants without LIS compared to HC. PSD with standard error mean (**e**) showed a steep overall decline in CLIS compared to HC, non-LIS, and LIS, with higher values in slow frequencies (1–8 Hz), a slowing of alpha peaks in the delta-theta range, and a power slope in the higher frequencies (10–40 Hz). Source data are provided in Supplementary Data 1.

Among the ten CLIS participants, EEG recordings of three patients from a longitudinal study^54^ were analysed. Participant #6 (male, 40 years old; 20 sessions over a 20-month period); participant #9 (male, 24 years old; 13 sessions over a 12-month period); participant #11 (male, 35 years old; 19 sessions over a 16-month period). In general, the three sets of EEG sessions show very similar pattern to those of the ten CLIS participants compared with healthy controls: higher mPLE, lower mLZC, lower cvPLE, and higher cvLZC (Fig. 5a–d). However, it is also possible to identify a trend toward progressive differentiation in the PLE and LZC results of healthy controls from the time that the patients reached a complete locked-in state. On the date of the first EEG recording, P#6 had already been in CLIS for five years, P#9 for one year, and P#11 began the transition to CLIS during the recording period. Although P#11 has results that are still close to the average of healthy controls (in particular mPLE, mLZC, and cvLZC), P#9 and P#6 progressively deviate from healthy controls. This is also visible in the PSDs of the three CLIS participants (Fig. 5e). While P#11 presents a power spectrum close to the average of the healthy controls (although we can already notice a greater inclination of the scale-free structure of that PSD), P#9 and P#6 show a progressive shift of power towards delta and theta ranges with residual alpha peak activity and a loss of power in the faster frequencies. Finally, a similar progressive trend can be seen regarding the variability of the values of the three CLIS participants between all sessions, whereby P#6 (longest time in CLIS) showed the highest standard deviation (P#6 SD: mPLE = 0.213; cvPLE = 0.058; mLZC = 0.033; cvLZC=0.033; P#9 SD: mPLE = 0.170; cvPLE = 0.017; mLZC = 0.032; cvLZC = 0.015) and P#11 (shortest time in CLIS) the lowest (SD: mPLE=0.075; cvPLE = 0.026; mLZC = 0.019; cvLZC = 0.012) (see also below, Fig. 6). Taken together, these results suggest that PLE and LZC are robust metrics of the temporal signatures of the EEG that can discriminate the severity of the changes in arousal within and between patients with CLIS. PLE and LZC may be clinically significant indices of disruptions to consciousness and have diagnostic and prognostic value.

### CLIS vs. LIS vs. non-LIS vs. healthy controls

This unique group of participants with ALS was crucial in identifying differences between healthy controls and people with ALS, but still capable of moving to some degree (non-LIS). A single participant with ALS in locked state (LIS) was unable to move but still able to communicate with the eyes, and two participants with ALS in complete locked state (CLIS) whose eye movements no longer allowed reliable communication for approximately two years^27^ were included in the following comparisons. Non-repeated measures tests did not show statistically significant differences in mPLE, mLZC, and cvPLE between healthy controls (*n* = 23) and non-LIS (*n* = 14), except for cvLZC (*p* < 0.05). In addition, one individual with LIS showed values of mPLE, mLZC, cvPLE, and cvLZC in the same range as ALS without LIS and healthy controls (Fig. 6a–c). Statistical comparisons using a *Z* test was performed on all values of the LIS participant. In all cases, the values for the LIS patient were *Z* < 1.96 (*p* > 0.05) from healthy controls (mPLE *z* = −0.012; mLZC *z* = 0.961; cvPLE *z* = −0.111; cvLZC *z* = 0.526) and non-LIS patients (mPLE *z* = 0.432; mLZC *z* = 0.242; cvPLE *z* = −0.454; cvLZC *z* = 1.659). By comparison, the two CLIS patients demonstrated the same trend as the CLIS dataset described above (Figs. 4, 5) with higher mPLE and cvLZC, and lower mLZC and cvPLE compared to HC and non-LIS. Again, this was statistically using a *Z* test for the two CLIS participants, showing *Z* > 1.96 (*p* < 0.05) from healthy controls, except for cvPLE (mPLE *z* = 4.694; 4.546; mLZC *z* = −3.120; −3.750; cvPLE *z* = −1.693; −1.801; cvLZC *z* = 6.625; 5.565), and non-LIS (mPLE *z* = 4.294; 4.172; mLZC *z* = −2.650; −3.096; cvPLE *z* = −1.749; −1.837; cvLZC *z* = 9.980; 8.534). The topographic maps for the difference between the mPLE, cvPLE, mLZC, and cvLZC groups are represented in Supplementary Fig. 4. The differences between the two CLIS participants and the other groups (healthy controls, non-LIS participants, and the single LIS participant) correspond well to visual inspection of the PSD (Fig. 6e), whereby the CLIS condition showed a generally steeper decline in PSD, with higher values in slow frequencies (1–8 Hz), with a slowdown and a shift in the alpha peak towards lower frequencies and a negative slope in higher frequencies (10–40 Hz). Taken together, these results suggest that PLE and LZC can discriminate between patients with LIS and CLIS, and between non-LIS and CLIS, and may be clinically significant indices of disruptions to consciousness and have diagnostic and prognostic value.

### Intra-subject fluctuations in a single CLIS case

**Fig. 7:**
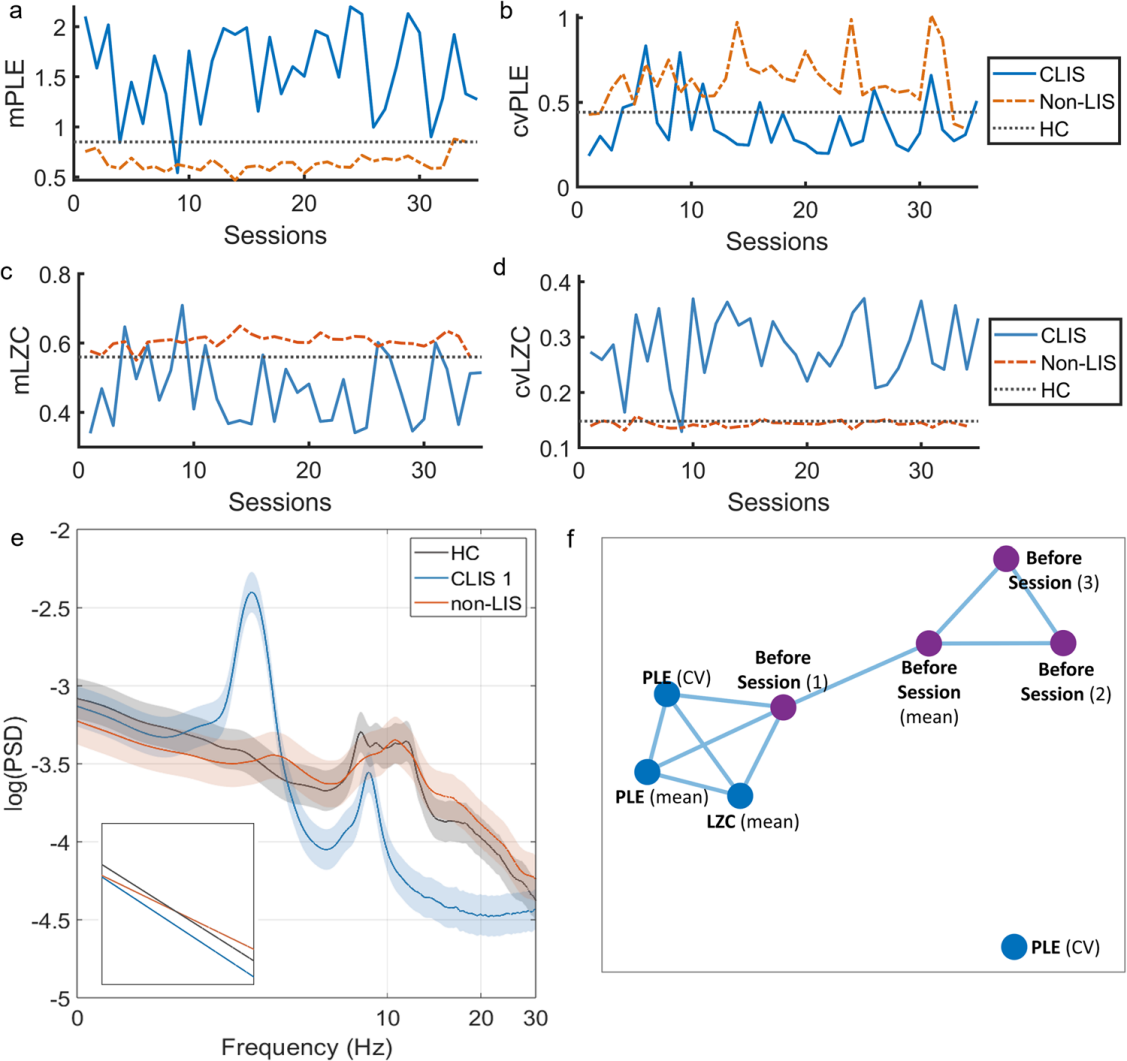
Longitudinal evaluation of PLE and LZC in a CLIS participant and a non-LIS participant from the ALS dataset (fluctuations between different sessions). The mean PLE (**a**) and LZC (**c**) distributions, and their respective coefficients of variation (**b**, **d**) are depicted over sessions for the CLIS and non-LIS participants, as well as for the mean of the healthy controls group (as multisession records in controls are missing). An increase for mPLE (with a decrease for cvPLE) and a decrease for mLZC (with an increase for cvLZC) are observed for the CLIS participant as compared to the non-LIS participant and the average of the HC. Moreover, fluctuations between sessions in the CLIS patients were detectable in all measurements. PSD with standard error mean (**e**) showed a power spectrum close to the mean of HC for the patient with ALS without LIS. The PSD of the CLIS participant showed a generally steeper decay compared to HC and non-LIS, with higher power at slow frequencies (1–8 Hz), a slowdown and a shift in power in the delta-theta range with residual peak alpha activity, and a loss of power at higher frequencies (10–40 Hz). The correlation network (**f**) represents the dynamic variables of the brain through the nodes of the networks, while the significant Spearman rho correlations between them (*p* < 0.05) are represented by the blue links. Three measures derived from the EEG recordings (mean and CV of PLE and LZC) show a statistically significant correlation with the behavioral status assessed immediately after the recording. This association is lost in subsequent evaluations. Source data are provided in Supplementary Data 1.

Furthermore, a series of 35 EEG recordings from one of the two CLIS participants in this dataset (female, 64 years old, named here as ‘CLIS 1’) were analysed, as well as a series of 34 EEG recordings from one of the ALS non-LIS participants (male, 59 years old) as a control for the analysis of intra-subjective variation. The multisession EEG analysis of the female participant in CLIS confirmed the results highlighted in Figs. 4–6, that is, PLE and LZC (both mean and coefficient of variation) in CLIS differ significantly from HC and non-LIS. Furthermore, this CLIS participant showed higher intra-subject variations in mPLE and mLZC during the sessions compared to the non-LIS participant (mPLE: CV = 0.276 vs. 0.133; mLZC: CV = 0.214 vs. 0.033) and also to the inter-subject variation among healthy participants in mLZC (mLZC: CV = 0.281 vs. 0.105). This higher intra-subject variation in CLIS is represented by fluctuations in mPLE, mLZC, and the cvPLE, and cvLZC values session after session (Fig. 7a–d). Furthermore, cvPLE and cvLZC (Fig. 7b, d) show that the degree of fluctuation of PLE and LZC within particular recordings is higher compared to controls and the non-LIS patient, that is, there are periods with high values of stability and periods with low stability (particularly in LZC). These differences between the CLIS participant and the non-LIS participant and, conversely, the similarities between HC and non-LIS, are also evident in the PSD (Fig. 7e). While the grand-average PSD of the non-LIS sessions is close to the average of the healthy controls, the grand-average PSD of the CLIS sessions shows an evident slowdown and shift of the power towards the delta-theta range with residual alpha peak activity and a reduced power in faster frequencies. Topographic maps also showed global significant changes between CLIS and non-LIS in mPLE, cvPLE, mLZC, and cvLZC (see Supplementary Fig. 4). Taken together, these case studies suggest that PLE and LZC can index arousal over time between patients CLIS and non-LIS patients.

### CLIS—from brain dynamics to behaviour

Our results thus far show that there is great variability of CLIS patients (in particular long-term CLIS) as compared to healthy controls, and non-LIS patients (see Figs. 5, 6). However, it remains to be demonstrated whether the dynamics measured by PLE and LZC are related to behaviour (and therefore symptom severity) in these patients. As previously mentioned, it is extremely difficult to assess the behavioural capabilities in these groups of patients. Indeed, CLIS is diagnosed when there is complete bodily immobility and unreliability of eye movements to communicate. Thus, there may still be residual eye movements that are unreliable for the purposes of communication. An assessment of overall state of vigilance was attempted using a scale where 0 = appears unconscious, 5 = appears fit and fully conscious (see Methods). The evaluation was carried out after the resting state session (5 min), and before starting each brain-computer interface session (‘Before session 1’, ‘Before session 2’, ‘Before session 3’, 12 min each). From these behavioural data and the measures of brain dynamics obtained from EEG (mean and coefficient of variation of LZC and PLE), we evaluated their possible inter-relationships. To do this, we generate a correlation matrix with all the variables involved (see Fig. 7f). In this approach, individual variables constitute nodes, and the relations between them constitute the edges. In this study, the relationships were evaluated using the Spearman test (due to the nature of the behavioural data and the lack of a priori hypothesis on the linearity of the relationship), showing only correlations between the variables that were significant. For visualisation, we used a direct application of the Fruchterman-Reingold algorithm^55^.

Interestingly, we found a significant correlation between PLE (mean and CV) and ‘Before session 1’ (i.e., the test values just after recording the resting state). Similarly, a significant correlation was found between LZC (mean) and ‘Before session 1’. These associations are lost in subsequent evaluations. The results show that, despite complete paralysis due to late-stage ALS and an inability of CLIS patients to communicate via eye movements, EEG dynamics were associated with the clinical-behavioural status of this patient.

See Table 1 for a summary of the main results described above from all the datasets analysed (mean, standard deviation, and statistical comparisons).

### Simulated signals: CLIS within the dynamic space of all possible PLE and LZC relationships (and values)

**Fig. 8:**
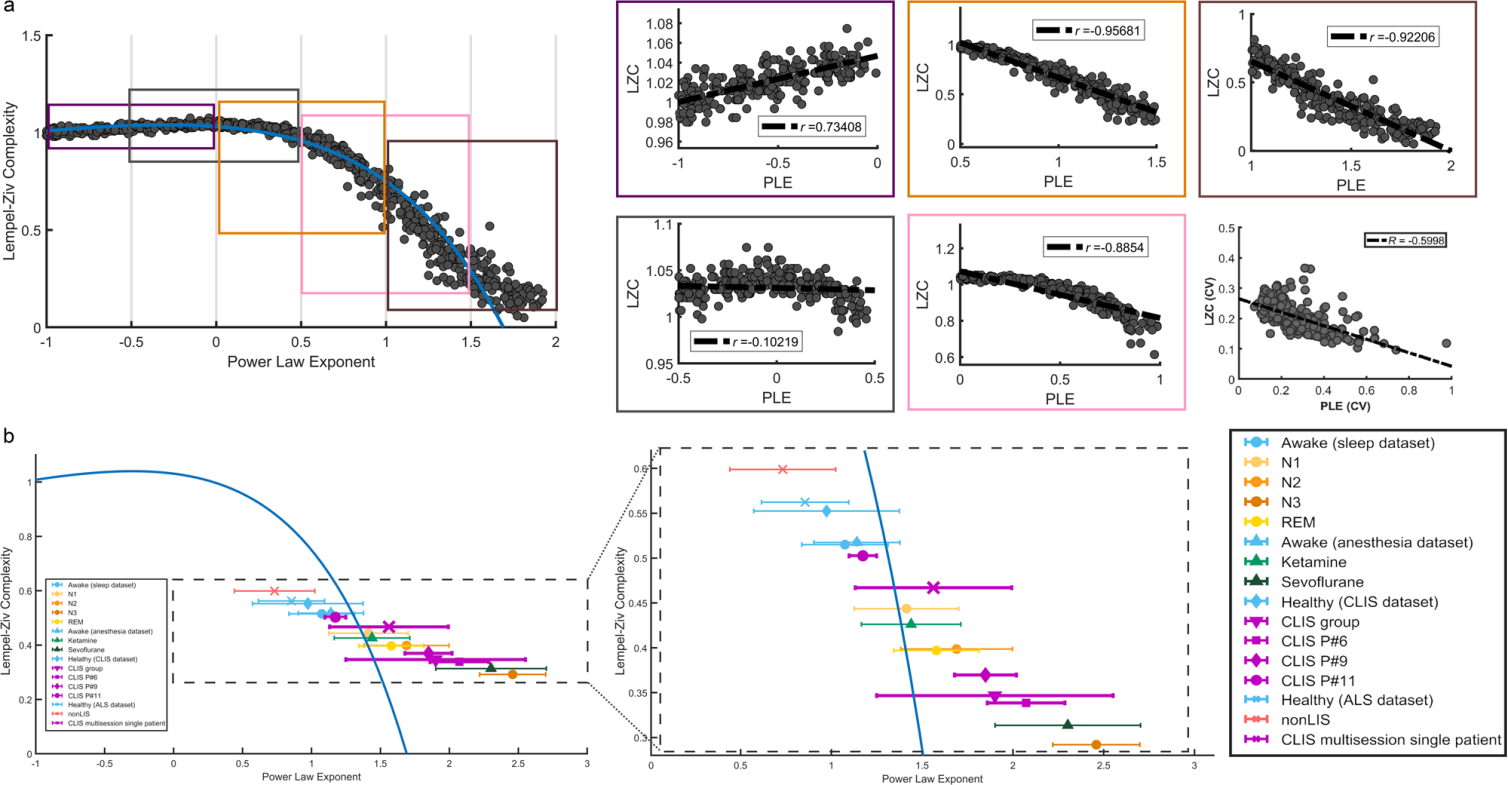
Relationship between LZC and PLE in empirical and simulated data. LZC and PLE are computed using synthetic data (**a**). For that purpose, 1000 brown, white, and pink noise signals were generated by varying the noise power decay factor with frequency. LZC and PLE calculated on these signals show a non-linear relationship between both measurements. In the empirical data, error bars were used to represent the mean of PLE and LZC as well as the standard deviation of PLE (**b**). Patients with CLIS patients from the different datasets show a greater variance in PLE values. Control participants are grouped around lower PLE values (PLE around 1, that is, pink noise). On the contrary, participants with altered alertness generally show higher PLE values and lower LZC values.

Although, in theory, any pair of LZC and PLE values is possible, brain dynamics exhibits a specific relationship between them. To analyse this relationship in a dynamic range larger than those provided by the empirical data, and at the same time, to provide a framework to analyse the space of brain operation (i.e., the ‘degree or level of arousal/wakefulness’) in which the different groups and states are located, a simulation was performed using artificially generated signals. To do this, 1000 synthetic noise signals were generated by varying the noise power decay factor with frequency (see Çatal et al. 2022 for details^56^)This decay factor was varied between a slope equal to zero (white noise) and a slope equal to −2. For each of the synthetic signals, LZC and PLE were calculated following the same procedure as with the empirical data (see Fig. 8a). These signals are intended to simulate the fractal component of the EEG^45^. The non-linear relationship between LZC and PLE follows an exponential trend (blue line). The LZC and PLE values (as well as PLE variability) of all groups in each dataset were depicted on such an exponential trend (Fig. 8b). Except for the non-LIS group, all healthy control groups were situated in higher LZC values and lower PLE compared to different states of reduced arousal. Interestingly, the CLIS groups generally showed a greater variance compared to the other groups (except perhaps for patient P#11, who, as seen above, was entering CLIS during the EEG recording phase).

Regarding the relationship between cvPLE and cvLZC using all available empirical data, a moderate/strong (negative) relationship was observed between both measures (Pearson’s *R* = −0.6). In other words, for high LZC fluctuation values, the corresponding PLE fluctuation values are low. Furthermore, low LZC fluctuation values seem to imply higher PLE fluctuations; however, this is less evident because the slope of the regression line is shallow (~0.22). These results suggest perhaps that large LZC fluctuations appear in ‘reduced’ arousal. However, caution must be exercised when interpreting these indirect results to avoid speculation.

## Discussion

We demonstrate how brain dynamics, measured by PLE and LZC, can indicate neural alterations and fluctuations within the state of CLIS compared to healthy participants and ALS patients with LIS/non-LIS conditions. When comparing these findings with the reference groups (sleep and anaesthesia), altered and variable temporal dynamics in the EEG-based PLE and LZC suggests fluctuating levels (*n.b*., not necessarily the absence) of arousal in CLIS. Furthermore, the results suggest that the longer a patient is in CLIS, the more altered the condition becomes, both in terms of the degree of fluctuation and overall state of arousal. Unfortunately, alterations in awareness or experiential consciousness cannot be directly inferred with these methods; neither in CLIS nor in the control groups. Since consciousness can be examined by different dimensions and characteristics^57^, for example, arousal and awareness, which are two classic clinical distinctions^32,53^, here, we consider arousal as the overall state of alertness or wakefulness. Having validated measures of neural dynamics, including PLE and LZC in two distinct states of reduced arousal (sleep and anaesthesia) parametrically along a graded spectrum (low: N1, REM, ketamine; high: N2, N3, sevoflurane), here, we show how in CLIS patients shift toward slower frequencies (high PLE) and lower signal complexity (low LZC), especially in patients with long-standing CLIS. Furthermore, patients with CLIS at both the group and individual levels show fluctuating arousal levels indicated by high intra-sessional variability (over the course of each EEG session), intra-subject variability (within the same individual, over different sessions), and inter-subject variability (between different CLIS patients). Taken together, we demonstrate that measures of brain dynamics such as PLE and LZC can serve to index vigilance states, which may indicate reduced and/or unstable states of arousal and wakefulness in CLIS. We, therefore, propose that intra-subject variability in PLE and LZC may be suitable biomarkers of fluctuations in arousal/vigilance over time within and between individuals^35,58^.

In terms of the relationship between brain dynamics and arousal levels, we show various degrees of increased PLE and decreased LZC in a wide range of stages where arousal is altered (e.g., ketamine, REM), moderately reduced (e.g., N1, N2), or strongly reduced, if not absent (e.g., N3, sevoflurane). This is in line with previous studies showing analogous changes in reduced states of wakefulness in both fMRI and EEG^22,38–40,42,51,59–66^. Our findings advance knowledge in this area in three important ways: First, these results show parametric changes in PLE and LZC during increasingly reduced wakefulness. Second, these results show the negative nonlinear relationship of LZC and PLE with changes in information complexity. This pattern is further supported by our simulation data (Fig. 8). Third, our findings indicate that the relationship of cvPLE and cvLZC exhibits different tendencies than the relationship of their means. Finally, we demonstrate high accuracy (above 90%) in predicting vigilance states, i.e., presence vs. absence of alertness, by PLE and LZC in almost all the comparisons. Together, this provides evidence that PLE and LZC of the EEG can be used as indices of different states of vigilance, which most likely correspond to different levels of arousal.

These findings make PLE and LZC suitable for estimating possible levels of arousal in CLIS at both group and individual levels. Our larger CLIS group showed a tendency towards high PLE and low LZC similar to reduced arousal in sleep and anaesthesia (see Fig. 8). This suggests that these participants are characterised by altered arousal. However, this does not appear to be a stable over time; as we observed alterations and fluctuations both within session, and between sessions in both PLE and LZC in the CLIS participants (see Fig. 8, the two CLIS datasets—group and individual participant—show the highest standard deviation values). Specifically, as shown in Fig. 5, the multisession EEG of the three participants in CLIS showed progressive alteration of PLE and LZC (especially mPLE, mLZC, and cvLZC) over time. In addition, PLE and LZC can serve as indices of CLIS-related severity; P#6 had been in CLIS for 5 years at the time of data acquisition and is distinct from controls. P#9 had been in CLIS for 1 year at the time of data acquisition, is also different from controls, but not to the same extent as P#6. On the other hand, P#11 entered CLIS during the data acquisition period and shows values closer to controls. A similar pattern was observed for PSD, which suggests that CLIS could also be related to a shifting of the alpha peak towards the theta-delta range (Fig. 5e).

The above findings could lay the groundwork and can serve to guide future directions for research. First, the progressive alteration in brain dynamics across the three participants suggests a trend that could be related to the years spent in CLIS. This could mean that patients with ALS-CLIS undergo a progressive alteration/reduction in arousal. Second, the fact that PLE, LZC, and PSD of P#9 (CLIS for 1 year) are more similar to P#6 (CLIS for 5 years) than to P#11 (in a transition state from LIS to CLIS) suggest that the time of alteration of brain dynamics (and arousal) may occur within a relatively short time from the diagnosis of CLIS. This implies the need to identify clinical and rehabilitation practises that seek to normalize these dynamics. Third, such alterations could represent one more piece that would help explain the hypothesis of ‘extinction of goal-directed thinking in complete paralysis’^11^. Indeed, alteration of arousal, is likely to prevent effective use of BCI in CLIS, perhaps at all, therefore communication attempts could be directed to times when arousal levels fluctuate to more normal values. Interestingly, the participant in CLIS #11 (Fig. 5), who spent less time in CLIS than the other two participants, who showed similar PLE and LZC values to the healthy control group, is the same patient who has been able to communicate with an innovative, yet invasive BCI, as recently demonstrated by Chaudhary and colleagues^9^. Thus, deciphering these fluctuations through PLE and LZC could be instrumental in establishing more objective measures for brain-computer interface communication in patients with CLIS^9–11,67^. In another study, several attempts at BCI communication were made with the CLIS participant presented in Fig. 7, with poor results probably due to the high instability of brain dynamics (e.g., fluctuations of alpha rhythm and power increase in the theta range^15,20^) or due to impaired cognitive abilities^19,30^. However, these hypotheses are based primarily on only three individuals, so further investigations are warranted.

Finally, through simulation data and the comparison with EEG data, we show the relationship between PLE and LZC. Specifically, the extreme shift towards slow frequency power and the loss of power in the high frequencies (high PLE) during the presumed reduced arousal/wakefulness relates to decreased signal complexity (low LZC) in a non-linear way. Changes in information processing may be driven by dynamic changes in the ratio of slow:fast frequencies during degraded, reduced, or absent levels of arousal. However, caution is warranted in interpreting these patterns, as our data do not allow us to test for causal relationships^44^.

Our findings may have important clinical and ethical implications. Measurement of brain dynamics, such as PLE and LZC, can serve as indices of the degree of arousal along the full continuum of increased and decreased arousal states. This could offer the opportunity to diagnose the presence or absence of BCI capability, particularly in CLIS, which, until now, has proven elusive. These findings may also provide a major steppingstone for developing new BCI to enable communication. For instance, either brief periodic or online analysis of brain dynamics in the resting-state of EEG through PLE and LZC could provide useful information to identify the most appropriate time of the day for treatments (e.g., physical therapy, neurorehabilitation) and communication attempts.

CLIS represents a sort of extreme condition in which consciousness can be maintained to some extent (with varying degrees of alteration, as suggested by this study), but without the possibility of evaluating it directly. Neuroscientific research based on theories of consciousness such as Integrated Information Theory (IIT) and Temporo-spatial Theory of Consciousness (TTC) could shed light on this issue^67–70^. Indeed, according to IIT and TTC various levels of spatiotemporal complexity of brain activity (measured here through LZC) can be associated with different states of consciousness. TTC considers LZC an index of the degree of signal compression as one key feature of the spatio-temporal repertoire of brain’s spontaneous activity, which is fundamental to information processing and the formation of the contents of consciousness^71^. In this sense, the perturbational complexity index (PCI) interpreted by IIT as a marker of the capacity to generate integrated information, i.e., consciousness according to the theory, is a normalised version of the LZC triggered by a direct Transcranial Magnetic Stimulation^40^. Furthermore, TTC considers a balanced (e.g., between slow and fast frequency power) temporal structure of neural dynamics (assessed through PLE) to be a necessary predisposition for arousal^69^. This temporal structure is altered or lost, during anaesthesia or sleep. In addition, the Global Neuronal Workspace Theory (GNWT)^72,73^ focusses mainly on global spatial recruitment of the whole brain through input from the dorsolateral prefrontal cortex and on event-related activity such as P3. The current study did not explicitly test for such global spatial recruitment, as clinical EEG does not provide adequate spatial resolution. However, the data show that there is global recruitment of the relative balance between different frequencies/timescales as measured by PLE and LZC. The data show that a relative balance of different frequencies is key for maintaining a conscious state, while their imbalance with an abnormal shift towards the slower frequencies leads to the disruption of consciousness. Such global temporal or dynamic recruitment of different frequencies in terms of their balances is a key feature of the TTC when assuming temporo-spatial nestedness as the main mechanisms related to level/state of consciousness^69^. Hence, the TTC complements here the GNWT in the need for global recruitment in the dynamic, and thus temporal, domain.

As for the limitations of this study, we investigated the temporal dynamics of the resting state using EEG. When not exposed to input from the external environment, or engaged in a specific task, the resting state exhibits its own spontaneous temporal dynamics, which may serve as an index of the capacity to process input from the environment and for cognition^74,75^. That said, PLE and LZC can only be indirectly related to an individual’s ability to interact with the outside world and communicate. An investigation of task states with BCI is needed to provide a more direct relationship between the level of stability of alertness in patients with CLIS and their cognitive ability to communicate. Thus, from the analysis of the resting-state alone, we cannot predict whether some cognitive task or external stimulation could limit the alteration of brain dynamics in CLIS and consequently improve the arousal state. However, as previously stated, the use of brain dynamics measurements, such as PLE and LZC, might indicate the best time to initiate BCI communication attempts. A future hypothesis that will be tested is that the resting-state before the task strongly influences cognitive performance, as other studies suggest^36,45,58,76–78^. However, it is important to note that although high BCI performance indicates a good level of consciousness, a low or absent BCI performance does not necessarily imply a low or absent consciousness. The patient may not want to answer, questions may be misunderstood, or the BCI device might have low precision^79^. Even in the case of a strong correlation between neuronal activity and state of consciousness, we should identify which dimensions of consciousness are involved (e.g., arousal, awareness, cognitive accessibility, etc.)^32,53^ and what degrees/levels of consciousness (despite ongoing debate surrounding the concept of ‘level of consciousness’^80^). Indeed, there may be cases of dissociation between these dimensions, such as arousal without awareness (similar to the vegetative state), levels of awareness/wake-like content of consciousness without arousal (similar to dreaming), arousal, and awareness but with cognitive deficits. Finally, as is often the case with unresponsive patients, it is not possible to directly infer the state of consciousness of patients in CLIS, due to the absence of any detailed first-person report or behavioural analysis. The fact that PLE and LZC in patients with CLIS are similar to the values of altered states of wakefulness such as sleep (N1, REM, N2, N3) and anaesthesia (ketamine, sevoflurane) does not necessarily imply that their state of consciousness is identical to these, in particular in terms of awareness. In other words, identical values do not necessarily mean the same level of reduction or loss of consciousness. See, for example, some cases of ‘paradoxical consciousness’ related to delta and theta activity (Angelman syndrome, epilepsy, behavioural responsiveness during propofol anaesthesia, postoperative delirium, and states of dissociation from the environment such as dreams and psychedelic states)^81^, as well as the case of ‘degraded consciousness’ after tiagabine described by Darmani and colleagues^82^. Together with our data, we can reliably, albeit indirectly, infer that CLIS patients could be characterised by instability and arousal degradation, without necessarily identifying their conscious states.

Patients with CLIS cannot communicate with the outside world and for this reason, their state of consciousness is unclear. Here, our objective was to provide indices of their level of arousal by using measures of brain dynamics, such as PLE, and the degree of signal compression and pattern repetitiveness, such as LZC (as an index of the complexity of information processing). We show extreme changes in PLE and LZC in CLIS at both the group and intra-individual levels. This suggests degraded, reduced, or even absent arousal in these patients, particularly those with prolonged CLIS compared to our benchmark, namely other altered states such as sleep and anaesthesia. Furthermore, patients with CLIS show high degrees of intra-subject fluctuations in both PLE and LZC, suggesting an unstable fluctuating state of alertness or wakefulness rather than a stable reduced state of consciousness. Together, our findings demonstrate that brain dynamics as measured with PLE and LZC can serve as an index to diagnose the state of consciousness, including its fluctuations, and, at the same time, may also have prognostic value in CLIS. The actual level of these measures at specific points in time may therefore be used to determine the optimal timing for communication interventions through assistive technologies such as BCI.

## Methods

### Participants

Four datasets were analysed: (1) sleep dataset, (2) anaesthesia dataset, (3) CLIS-ALS dataset, and (4) ALS dataset (with various levels of motor impairment, see below). A description of each is given below and is summarized in Table 2.

**Table 2:** Summary of the main characteristics of each dataset.

	Sleep dataset	Anaesthesia dataset		CLIS-ALS dataset		ALS dataset			
		Ketamine	Sevoflurane	Healthy controls	CLIS	Healthy controls	non-LIS	LIS	CLIS
Participants	23	10	10	6	10	23	14	1	2
Age	26 ± 6.5	32.9 ± 9.5	41.4 ± 13.1	45 ± 14,8	47.1 ± 20.7	44.8 ± 7.5	58.5 ± 11.8	52	61.5 ± 3.5
Sex (m:f)	8:15	6:4	8:2	4:2	5:5	13:10	7:1 + 4 n.a.	0:1	1:1
Electrodes	11	256	256	16	1-20	121			
Sampling rate in acquisition (Hz)	512	1000	1000	500		500			
Sampling rate after resampling (Hz)	256	250	250	250		250			
Recording time (cut)	10 × 30 s epochs (5 min)	5 min	5 min	5 min		5 min			

*n.a.* not available.

### Sleep dataset

Twenty-three healthy adults (age = 25.95 ± 6.53 years, 15 women) were included in this study. All participants reported normal sleep patterns and were free of signs of sleep disorders, according to standard guidelines^83^, evaluated by a night of polysomnography (PSG). Participants performed a complete PSG using the Embla Titanium PSG system (Natus, San Carlos, CA) PSG system. The EEG, EOG, and EMG signals were acquired with impedances < 5 kΩ, at a sampling rate of 512 Hz, referenced to FPz. The EEGs were acquired using 11 gold-plated electrodes placed according to the conventional 10–20 system. The EEG signals were re-referenced offline to the average of the mastoid derivations for sleep stage scoring. Sleep stages (wake, N1, N2, N3, REM) were marked using RemLogic analysis software (Natus) following standard criteria^84^. See Fang et al.^85^ for a further detailed description of the dataset.

### Anaesthesia dataset

#### Ketamine

For the anaesthesia dataset, the effects of two different general anaesthetics, namely, ketamine and sevoflurane, were evaluated in two distinct groups of 10 participants each. To assess the effect of ketamine, resting-state EEG recordings (Ges300, EGI, USA) in ten right-handed surgical patients (age = 32.90 ± 9.48 years, 4 women), in awake conditions (5 min eyes-closed) condition using an electrode cap (HydroCel 130) with 256 electrodes. After 10 min of quiet rest, patients were asked to close their eyes and a 5 min EEG recording was taken in the awake state, acquired using Netstation 4.2 software (EGI, USA). Then, 1 mg/kg of diluted ketamine in 10 ml of 0.9% normal saline was infused for 2 min, until the Observer’s Assessment of Alertness/Sedation (OAA/S) scale was 0 (no response to trapezius squeeze). When the OAA/S score was 0, the ultrashort-acting opioid remifentanil (1 μg/kg) and the neuromuscular relaxant rocuronium (0.6 mg/kg) were administered for endotracheal intubation. After aesthetic induction, the diluted ketamine was infused again for 20 min (1 mg/kg/h). EEG data were acquired for 5 min, taking place 15 min after loss of responsiveness. All patients wore ear plugs to avoid disruptions from environmental noise. EEG acquisitions were made at a sampling rate of 1000 Hz, the electrode impedance was kept under 5KΩ, and bandpass hardware filters were set between 0.1 and 100 Hz with a notch filter at 50 Hz. All channels were referenced to Cz.

#### Sevoflurane

Similarly, the other 10 participants (age = 41.4 ± 13.1 years, 2 women) followed the same protocol, but under sevoflurane anaesthesia. In this case, 8% sevoflurane was initially administered in 100% oxygen 6 L/min, and when the OAA/S score was 0, remifentanil (1 μg/kg) and rocuronium (0.6 mg/kg) were administered for endotracheal intubation. After induction of the anaesthetic, the end-tidal concentration of sevoflurane was maintained at 1.3 MAC (2.6%). EEG data were acquired for 5 min, taking place 15 min after loss of responsiveness. The equipment and the EEG acquisition procedure were identical to those followed under the effects of ketamine. During the study period, electrocardiogram, non-invasive blood pressure, and pulse oximetry were monitored in these non-premedicated patients (see Supplementary Table 1 for further details).

### CLIS-ALS dataset

A total of ten late-stage ALS patients with CLIS were included (age = 47.1 ± 20.74 years, 5 women). CLIS was defined as the inability to communicate with eye movements or any other voluntary muscle with the use or non-use of eye trackers for more than 6 months. The same protocol was also carried out on six healthy controls in the awake condition (age = 45 ± 14,79 years, 2 women)^7^.

Furthermore, an EEG dataset was included from three of the ten participants with CLIS, with multiple EEG sessions:^54^ Participant #6 (male, 40 years old; 20 sessions over a 20-month period; data acquisition: May 2017—January 2019); participant #9 (male, 24 years old; 13 sessions over a 12-month period; data acquisition: June 2017—June 2018, 12 months); participant #11 (male, 35 years old; 19 sessions over a 16-month period; data acquisition: May 2018—September 2019). The EEG data were acquired for 5–17 min (reduced then to 5 min to standardise the data) with eyes closed for both the CLIS and healthy participants. EEG signals were recorded using a V-Amp amplifier and active electrodes (Brain Products, Germany). The placement of the electrodes followed the international 10-5 system, with reference and ground channels placed, respectively, on their right mastoid and forehead. Due to clinical needs, the number and position of the sensors were different between the patients, while they were identical between the healthy participants. For a more detailed description of the acquisition procedure and clinical conditions, see related studies^7,25,54^.

### ALS dataset

Fourteen non-LIS ALS patients (age = 58.5 ± 11.78 years, 9 men, 1 woman, 4 n.a.) with ALSFRS-R scores of 3–40 (min = 0, max = 48)^86^, a single female ALS patient (age = 52 years) in LIS (ALSFRS-R = 1), and two ALS patients (one male, age = 43; one woman, age=64) in CLIS (ALSFRS-R = 0) participated in the study (we believed it was appropriate to keep these two CLIS patients within this dataset and not in the ‘CLIS-ALS’ dataset due to differences in EEG settings and EEG equipment). Additionally, an EEG dataset with multiple recordings from one of the fourteen non-LIS participants (male, 59 years old, 34 sessions eyes open) and one of the two CLIS participants (female, 64 years old, 35 sessions eyes closed) were included. The EEG data were acquired for 5 min (eyes closed) using 121 active electrodes at a sampling frequency of 500 Hz (Brain Products GmbH, Germany). The placement of the electrodes followed the international 5–10 system, reference to the left mastoid. For a more detailed description of the acquisition procedure, see related studies^19,27,87,88^. The same protocol was performed on 23 healthy controls in the awake condition (age = 44.79 ± 7.47, 10 women).

In addition, regarding the female patient with CLIS, an assessment of overall state of vigilance was attempted using a scale where 0 = appears unconscious, 5 = appears fit and fully conscious. Through these residual eye movements, we attempted to determine from a behavioural point of view, her vigilance status (e.g., by checking the ability to keep the eyelids open, observing the frequency of eye movements, and whether the eyes tend to roll back). This evaluation was carried out at various stages of the visit during a brain-computer interface communication experiment, where the patient with CLIS had to try to answer personal questions by performing two cognitive tasks: (1) thinking of positive memories, and (2) subtracting numbers for 15 s^89,90^. The evaluation was carried out after the resting state session (5 min), and before starting each brain-computer interface session (‘Before session 1’, ‘Before session 2’, ‘Before session 3’, 12 min each).

### Ethics statement

All participants (or their legal guardians) gave their informed written consent before participating. This research was approved by the respective Universities/Hospitals depending on the origin of the dataset (sleep dataset: Western University Health Science Research Ethics Board; Anaesthesia dataset: Huashan Hospital, Fudan University; CLIS dataset: Medical Faculty of the University of Tübingen; ALS dataset: Max Planck Society Ethics Committee). This study was conducted in accordance with the Declaration of Helsinki guidelines.

### Pre-processing

Due to the diversity of the recordings from the four datasets used in the present study, different pre-processing procedures were carried out. For this purpose, the data were resampled after aliasing filtering to avoid possible biases due to the similar but different sampling rates during the acquisition. We also took special care in removing muscular and ocular artifacts. All preprocessing was applied using custom MATLAB scripts (The MathWorks, 2017b) and the EEGLAB toolbox.

#### Sleep recordings

In the case of the sleep dataset, 30-second length epochs were classified according to the particular sleep stage. However, due to the length of the EEG recordings (overnight polysomnography during nocturnal sleep), epochs labelled as movement/noise/unscored by a registered technologist were excluded from subsequent analyses. In the remaining epochs, a FIR filter between 0.5 and 45 Hz was applied to the data, as in the other dataset. In particular, sleep data show the main differences because of the duration of the recordings and the high nonstationarity of the signals compared to the other datasets. The sleep recordings have a duration of about 8 h, whereas the other datasets are only a few minutes in duration. In addition, the different stages of sleep that a person goes through at night are well known, with the stationarity of the EEG signals lower than during wakefullness^91^. These sleep stages have different characteristics in terms of the amplitude and frequency of the EEG signal^91^, so considering each sleep stage separately is imperative. The other main difference between datasets is the number of electrodes. Since sleep recordings are from standard polysomnography (PSG), the number of electrodes is lower than that of the other datasets.

#### Anaesthesia, CLIS, and ALS recordings

First, the sampling rate was downsampled to 250 Hz using EEGLAB’s resample function. Second, the continuous data were bandpass filtered from 0.5 to 45 Hz. The unused channels were then removed, i.e., peripheric channels, as well as channels related to ocular or heart movements. Next, clean_rawdata EEGLAB plugin was applied to remove flatline channels, low-frequency drifts, noisy channels, and short-time bursts from each EEG channel. The recordings were referenced to the average activity. Finally, stationary artefacts, specifically eye movements, muscular noise, and line noise were removed using ICA. The CLIS dataset has a low and variable number of electrodes due to clinical factors (from 1 to 20, and the majority of patients with less than 10 electrodes; for details^7,54^).

### Measurements

Brain dynamics and information processing in the brain can be measured in various ways. One measure captures the balance in the power of slow-fast frequencies, scale-free dynamics^92–95^, which can be measured by the PLE^38,39,61,96^. Importantly, rather than changes in a specific single frequency band, altered states of consciousness exhibit changes in the structure of the power spectrum that holds across the different frequencies; this is reflected in increases in PLE during sleep^38,61,66,97^, anesthesia^38,39,42^ and disorders of consciousness^38,60,98–100^. In addition to PLE, the LZC; a metric of how regular/repeatable, or diverse the EEG signal is over time^101–103^ was used. Importantly, changes in LZC (and its alternatives) have been associated with changes in state of consciousness (see Sarasso et al. for a detailed review^104^) such as sleep^40,43,105–108^, anesthesia^39,40,42,51,109^, and disorders of consciousness^39–41,98,99,110,111^.

### Spectral and PLE analysis

To estimate the PSD of the EEG data, the Welch method was calculated^112^. This method requires a split of the EEG time series into overlapped segments of length *L*. For our analysis, *L* was set to 3 times the sampling rate (e.g., 3 s), with an overlap of 50%. Then, the segments were smoothed using a Hamming window. The Fast Fourier Transform (FFT) was applied in an epoch-based way to obtain the modified periodogram. Finally, PSD was estimated by averaging all periodograms. This allows us to obtain an adequate resolution (two data samples per Hz) with an assumable increase in computational cost. PSD values represent the power of oscillatory neuronal activity across the frequency spectrum (see Supplementary Fig. 5 for a representation of sample of raw EEG data from each group, with the power spectral density of the given EEG session).

Once the PSD was computed, the PLE was obtained using custom Matlab scripts. For this purpose, the PSD representation was logarithmically transformed in both the frequency spectrum and the power spectrum range. Then, the slope of the PSD was estimated by computing linear least squares regression. Finally, the PLE of each was defined as the absolute value of such a slope. The average PLE across epochs and channels was used for further analysis. PLE values represent the extent of wide-band arrhythmic neuronal activity in the EEG. Thus, lower PLE values, that is, more flatness in the PSD function, are associated with more arrhythmic activity. The extreme is a white noise signal with a completely flat PLE.

It is worth noting that the PLE complements the PSD analysis by identifying differences in the temporal structure of the spectrum power (in a static way). Although the PSD shows the differences in the power spectrum in terms of absolute power at particular frequencies, the PLE instead highlights the specific relationship in power between slow and fast frequencies, showing how their balance is altered in certain states, e.g., in anaesthesia^39^. For this reason, the increase in power of slower frequencies is not always and necessarily associated with a higher negative slope of the PSD (i.e., higher PLE) and vice versa. For example, a PSD that shows high power at slower frequencies may be associated with a low PLE (flat slope) in the case of an increased power also at faster frequencies. On the other hand, a PSD that shows low power at slower frequencies may be associated with a higher PLE in the event of an excessive decrease in the faster frequencies. Importantly, PLE measures changes in the structure of the power spectrum across different frequencies rather than measuring changes in one specific single frequency band.

### LZC analysis

LZC is a nonlinear measure of complexity that estimates the rate of occurrence of distinct sub-sequences or patterns in a given time series^44,113,114^. To calculate the LZC, the discrete signal in the time domain, *x*[*t*], is binarized into a new sequence (*P*) using a threshold (*T*):

1 $$P=s\left(1\right),s\left(2\right),\ldots ,s\left(n\right)$$

where

2 $$s\left(i\right)=\left\{\begin{array}{c}0,{{{{{\rm{if}}}}}}x\left[i\right] \, < \,T\\ 1,{{{{{\rm{otherwise}}}}}}\end{array}\right.$$

After binarizing the signal, the sequence *P* is scanned from left to right and the complexity counter is increased by one unit every time a new subsequence of consecutive characters is encountered. Among the different thresholding options, we used a median split, since it has been commonly applied in previous studies due to its robustness to outliers^115^. In particular, we followed the following algorithm:^116^

1. Let *S* and *Q* denote two subsequences of P and *SQ* be the concatenation of *S* and *Q*, while sequence ${SQ}\pi$ is derived from *SQ* after its last character is deleted (*π* denotes the operation of deleting the last character in the sequence). Let $v\left({SQ}\pi \right)$ denote the vocabulary of all different subsequences of ${SQ}\pi$ . At the beginning, the complexity measurement is $c\left(n\right)=1$, $S=s\left(1\right)$, $Q=s\left(2\right)$, therefore ${SQ}\pi =s\left(1\right)$.
2. In general, $S=s\left(1\right),s\left(2\right),\ldots ,s\left(r\right)$, $Q=s\left(r+1\right)$, then ${SQ}\pi =s\left(1\right),s\left(1\right),\ldots ,s\left(r\right)$; if *Q* belongs to *v*(*SQπ*), then *Q* is a subsequence of *SQπ*, not a new sequence.
3. Renew *Q* to be $s\left(r+1\right)$, $s\left(r+2\right)$ and check if $Q$ belongs to $v\left({SQ}\pi \right)$ or not.
4. Repeat the previous steps until *Q* does not belong to $v\left({SQ}\pi \right)$ . Now $Q=s\left(r+1\right),s\left(r+2\right),\ldots ,s\left(r+i\right)$ is not a subsequence of ${SQ}\pi =s\left(1\right),s\left(2\right),\ldots ,s\left(r+i-1\right)$, so increase $c\left(n\right)$ by one.
5. Thereafter, *S* is renewed to be $S=s\left(1\right),s\left(2\right),\ldots ,s\left(r+i\right)$, and $Q=s\left(r+i+1\right)$.

This procedure is repeated until *Q* is the last character, being $c\left(n\right)$ the number of different subsequences in *P*. Finally, $c\left(n\right)$ is normalized to reflect the arising rate of new patterns in the sequence as follows:^102^

3 $${LZC}=\frac{c\left(n\right)}{n/{{\log }_{2}\left(n\right)}^{2}}$$

where *n* is the length of the time series.

### Sliding window

Since we were interested in characterising the time evolution of the measures explained above (LZC and PLE), that is, computing the temporal dynamics of the signal over time, we used a sliding-window approach. Hence, we use 1-second-length windows with 50% overlap as a trade-off between a sufficient number of windows and sufficient temporal resolution. To verify that the percentage of overlap did not influence the outcomes, and that the length of the chosen window is sufficient for the calculation of the parameters, we also used windows of 5 s duration with 90% overlap (results in the Supplementary Figs. 6–9). This approach verified that this procedure used a window length that was adequate to estimate the scale-free dynamics (PLE), and that the normalisation of the LZC was independent of the window length (see Eq. 3). Note that these two approaches result in the same number of windows. Furthermore, to confirm the PLE and LZC results, in the Supplementary Information, the autocorrelation window (ACW) was also calculated as a control analysis, which has been shown to be related to neural input processing and consciousness^38,74,75^ (Supplementary Figs. 6–9). After following the sliding-window procedure, the mean of the parameters across the sliding windows (mPLE and mLZC) was calculated, as well as the coefficient of variation (cvPLE and cvLZC).

### Statistics and reproducibility

Statistical analysis was performed with the Matlab ‘Statistics and Machine Learning’ Toolbox (version 2017b). Both to analyse the distribution of the values of each dataset (violin plots) and to study the distribution of the measurements calculated on the scalp (topographic plots), normality and homoscedasticity were assessed using the Kolmogorov–Smirnov test and Levene test, respectively. Given violations of the assumptions for parametric tests (normality and homoscedasticity), and the low number of participants in certain groups of specific datasets, non-parametric tests were employed. For pairwise comparisons, the Mann-Whitney U test and the Wilcoxon signed-rank test were used to assess comparisons between groups and within groups, respectively. In the specific scenario of the sleep dataset, a comparison was conducted among five distinct conditions (namely, wake, N1, N2, N3, and REM). Consequently, the Friedman test was employed in this particular case. Post hoc comparisons were performed after controlling for the false discovery rate (FDR)^117,118^.

For correlations, Spearman’s rho test was used since we do not have an a priori hypothesis about the type of relationship between the variables (i.e., linear or nonlinear relationship).

To further analyse the robustness of the results, a receiver operating characteristic (ROC) curve was computed for each dataset and measure (Supplementary Figs. 1–3, 6–8). The ROC curves illustrate the diagnostic ability of a binary classifier as its discrimination threshold is varied. To obtain these curves, the true positive rate (i.e., sensitivity) is represented against the false-negative rate (i.e., 1- specificity) at different thresholds. Finally, the area under the ROC curve (AUC) was calculated. It ranges from 0 to 1. The higher the AUC, the higher the robustness or performance of the binary classifier^119^.

### Reporting summary

Further information on research design is available in the Nature Portfolio Reporting Summary linked to this article.

## Supplementary information

Supplementary Information

Description of Additional Supplementary Files

Supplementary Data 1

Reporting Summary

## Data Availability

Source data used to generate figures are available as Supplementary Data 1. The raw data that support the findings of this study are available on request from the corresponding author, upon reasonable request. The data are not publicly available due to ethical requirements and privacy concerns.

## Code availability

Matlab codes are available at the following URL: https://github.com/Temporo-spatial/PLE_LZC.
