# Supplementary material for: Altered brain dynamics index levels of arousal in complete locked-in syndrome
Source: Commun Biol. 2023 Jul 20;6:757. doi: 10.1038/s42003-023-05109-1 (PMC10359418; doi:10.1038/s42003-023-05109-1)
Supplement: Supplementary file 1 — Supplementary Information [file 42003_2023_5109_MOESM1_ESM.pdf]

Supplementary Information

Altered brain dynamics index levels of arousal in complete locked-in syndrome

Federico Zilio[1], Javier Gomez-Pilar[2,3], Ujwal Chaudhary[4,5], Stuart Fogel[6,7], Tatiana Fomina[8], Matthis Synofzik[9,10], Ludger Schoels[9,10], Shumei Cao[11], Jun Zhang[11], Zirui Huang[12], Niels Birbaumer[13], Georg Northoff[7]

[1] Department of Philosophy, Sociology, Education and Applied Psychology, University of Padova, Padua, Italy.

[2] Biomedical Engineering Group, University of Valladolid, Valladolid, Spain.

[3] Centro de Investigación Biomédica en Red en Bioingeniería, Biomateriales y Nanomedicina (CIBER-BBN), Valladolid, Spain.

[4] BrainPortal Technologies GmbH, Mannheim, Germany.

[5] ALS Voice gGmbH, Mössingen, Germany.

[6] School of Psychology, University of Ottawa, Ottawa, Canada.

[7] Institute of Mental Health Research, University of Ottawa, Ottawa, Canada.

[8] Department for Empirical Inference, Max Planck Institute for Intelligent Systems, Tübingen, Germany.

[9] Department of Neurodegenerative Diseases and Hertie-Institute for Clinical Brain Research, University of Tübingen, Germany.

[10] German Center for Neurodegenerative Diseases (DZNE) Tübingen, Germany.

[11] Department of Anesthesiology, Fudan University Shanghai Cancer Center, Shanghai, China.

[12] Center for Consciousness Science, Department of Anesthesiology, University of Michigan Medical School, Ann Arbor, USA.

[13] Institute of Medical Psychology and Behavioral Neurobiology, University of Tübingen, Tübingen, Germany.

Corresponding author

Federico Zilio

Department of Philosophy, Sociology, Education and Applied Psychology, University of Padova, Palazzo del Capitanio 3, Padua 35139, Italy

federico.zilio@unipd.it

ORCID: 0000-0001-9220-7372

Supplementary Measurements

a. Supporting analysis – Autocorrelation window

The autocorrelation window (ACW) was computed in the Supporting Materials for each of the participants from the four datasets. For that purpose, custom scripts were developed to compute the ACW by measuring the full-width-half-maximum of the temporal autocorrelation function of each electrode, following the description provided by Honey and colleagues[1]. Autocorrelation was calculated using windows of 20 s-length with and overlap of 50%. The lag was set to 0.5 s since we observed in a previous study that the ACW values agreed for different lag values (ranged from 0.1 to 1 s)[2]. The full-width-half-maximum of the main lobe of each the autocorrelation functions was then computed for each epoch. ACW was estimated as the average of all the epochs for each electrode and condition. In order to reduce the number of comparisons and to minimize type I errors, a grand average across electrodes was performed. ACW values represent the extent of the periodicity of the EEG signal, whereby longer ACWs can be interpreted as greater stability of the frequencies over time. The length of the ACW can be seen, therefore, as an index that summarizes the degree of regularity of a signal, with longer ACW associated with more regular EEG oscillations. On the contrary, considering the extreme case, the autocorrelation of a white noise signal will have a peak in the origin, whereby the ACW, in this case, would be zero.

Supporting analysis

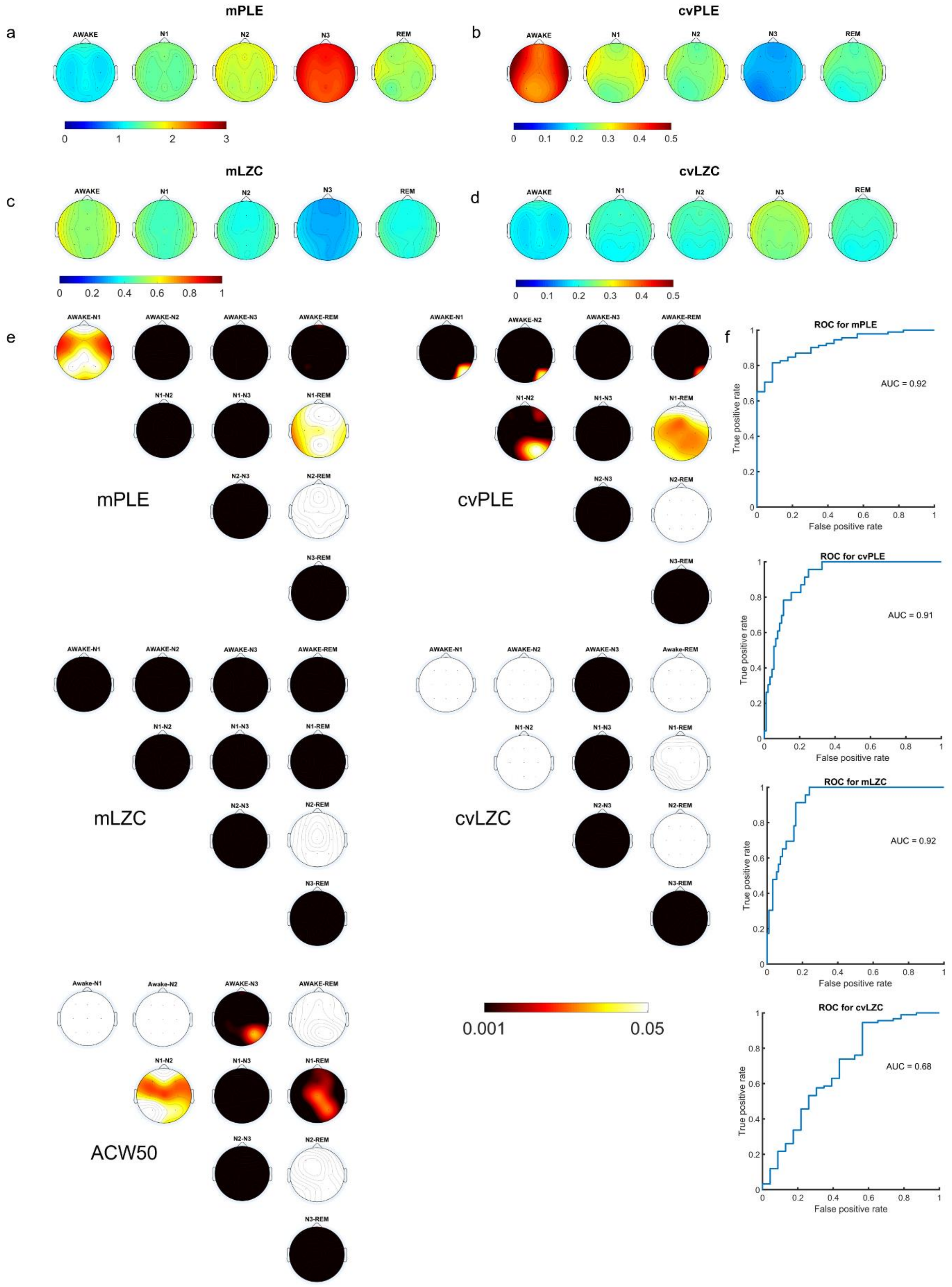

Supplementary Fig. 1: Sleep dataset. Topographical maps, comparisons of the topographical maps with FDR correction (Benjamini-Yekutieli), and Receiver Operating Characteristic curves (ROCs)

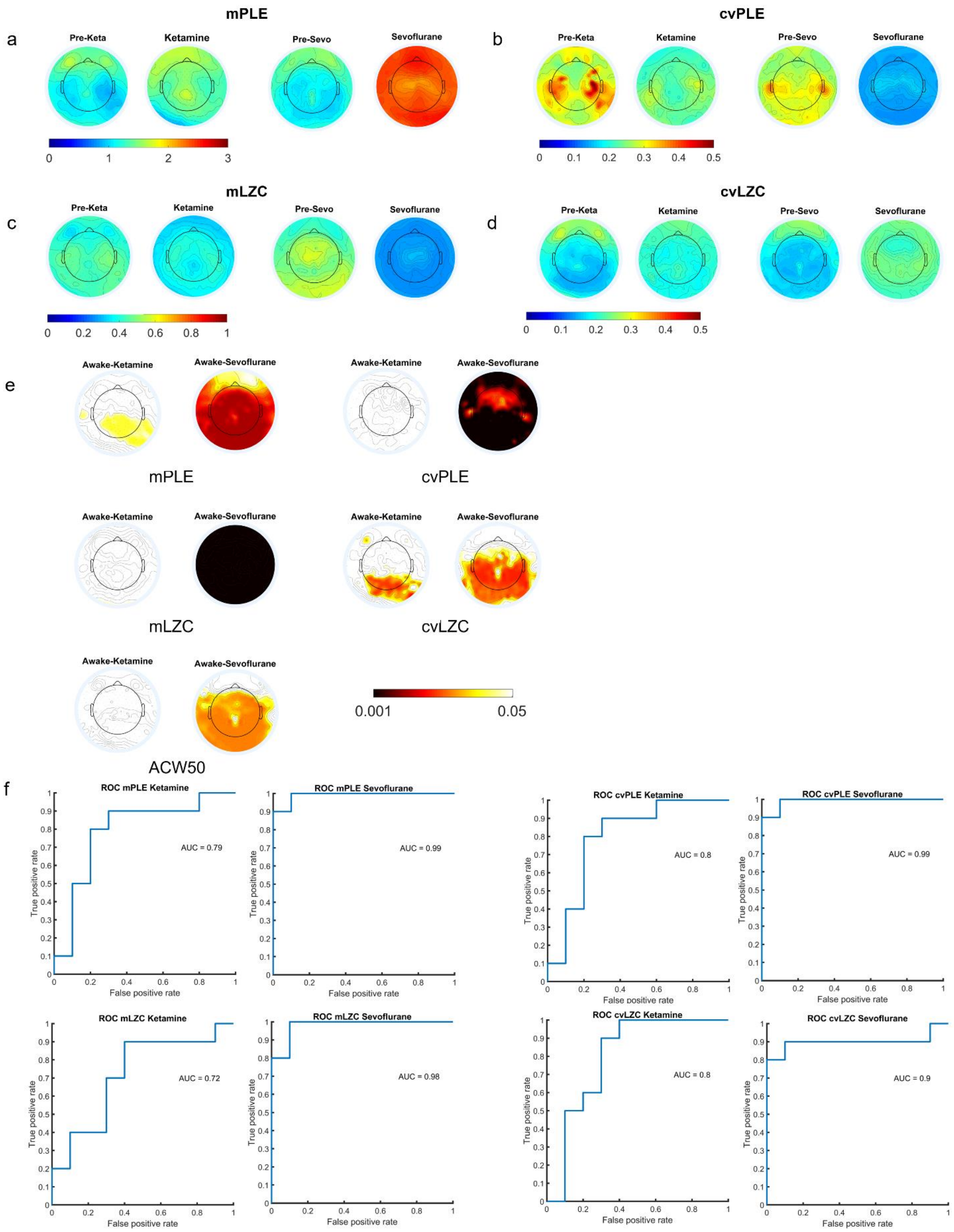

Supplementary Fig. 2: Anesthesia dataset. Topographical maps, topographical map comparisons with FDR correction (Benjamini-Hochberg), and Receiver Operating Characteristic curves (ROCs)

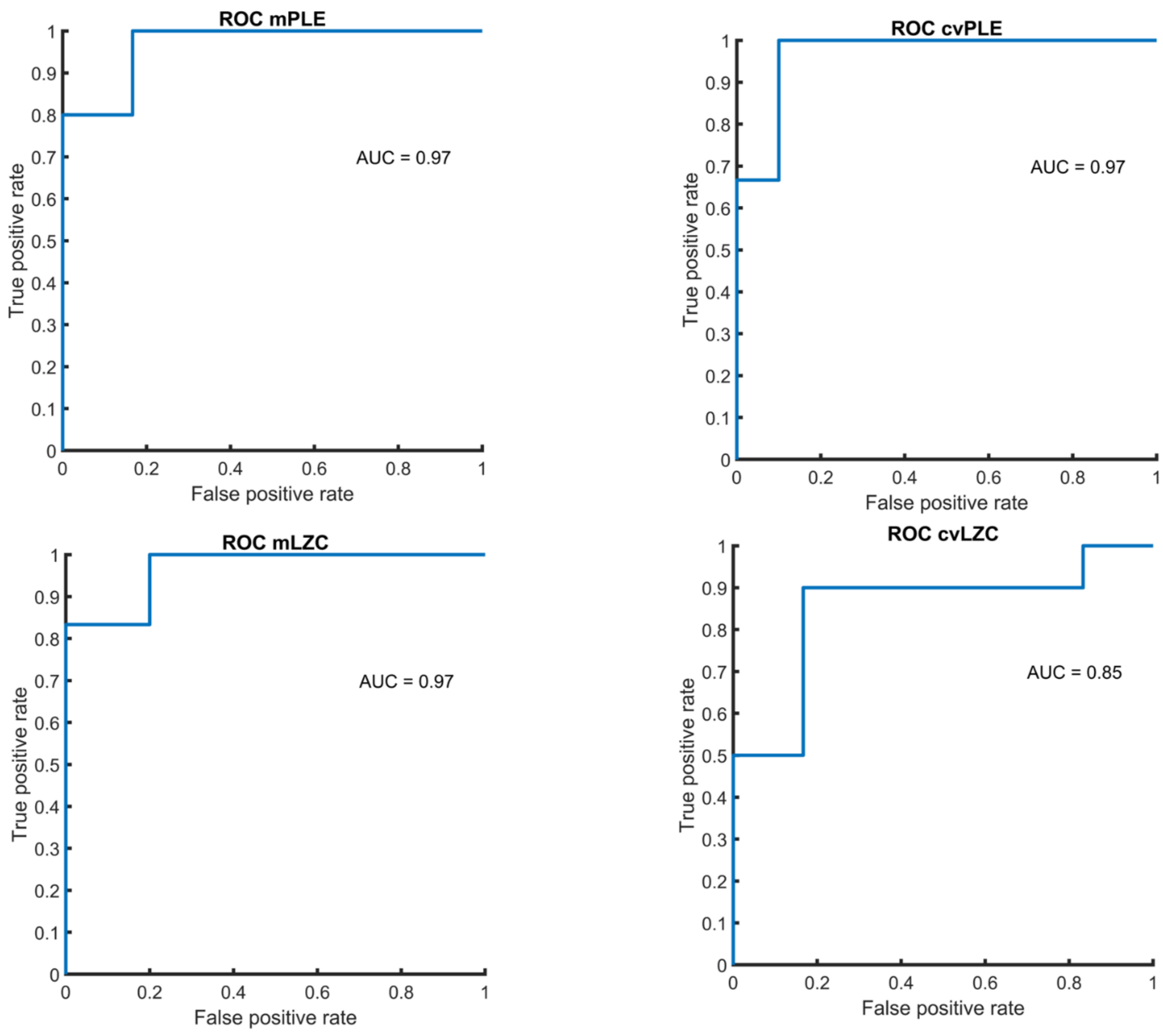

Supplementary Fig. 3: CLIS dataset (Healthy controls vs CLIS). Receiver Operating Characteristic curves (ROCs)

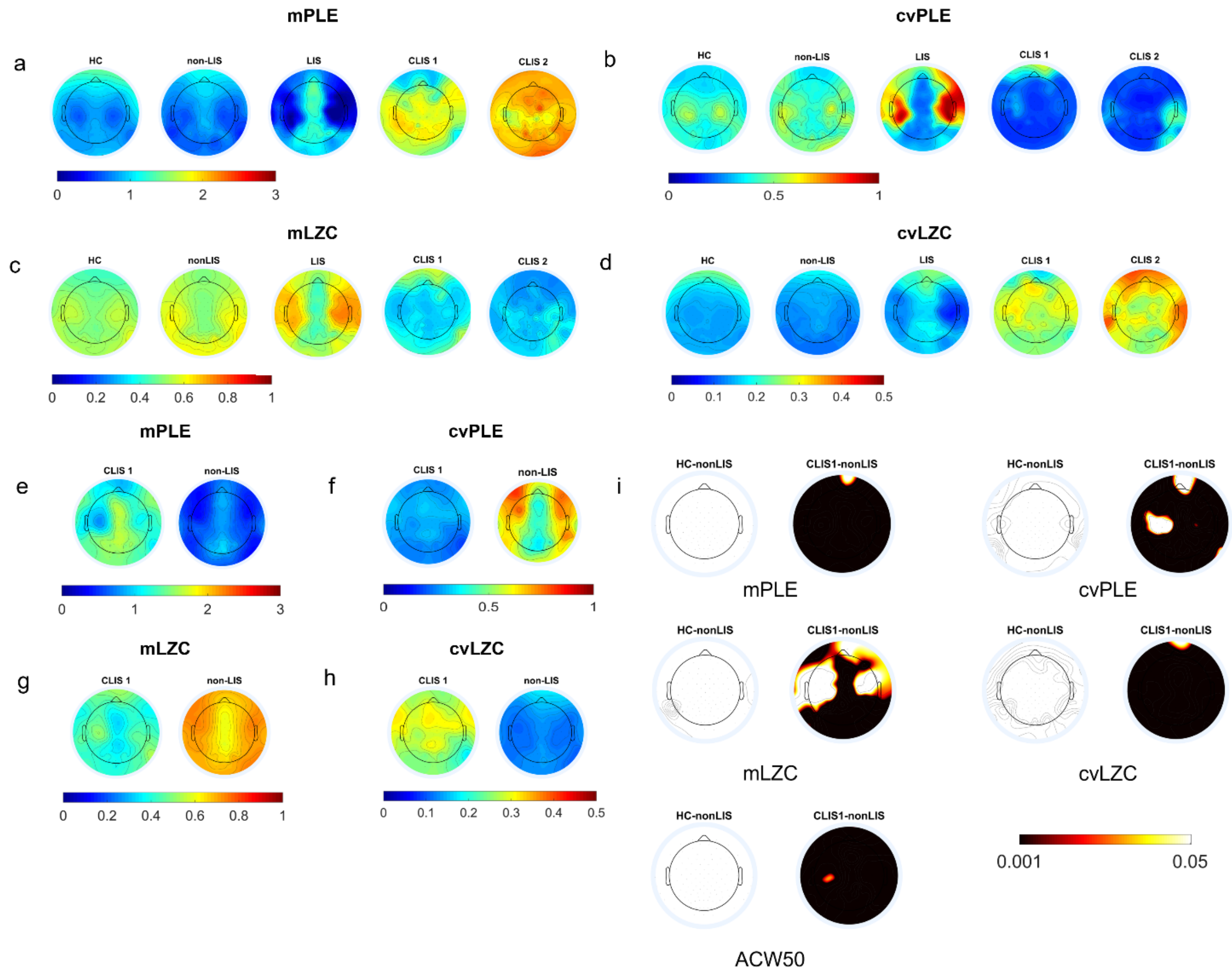

Supplementary Fig. 4: ALS dataset. Topographical maps, and CLIS vs. non-LIS topographical map comparisons with FDR correction (Benjamini-Yekutieli)

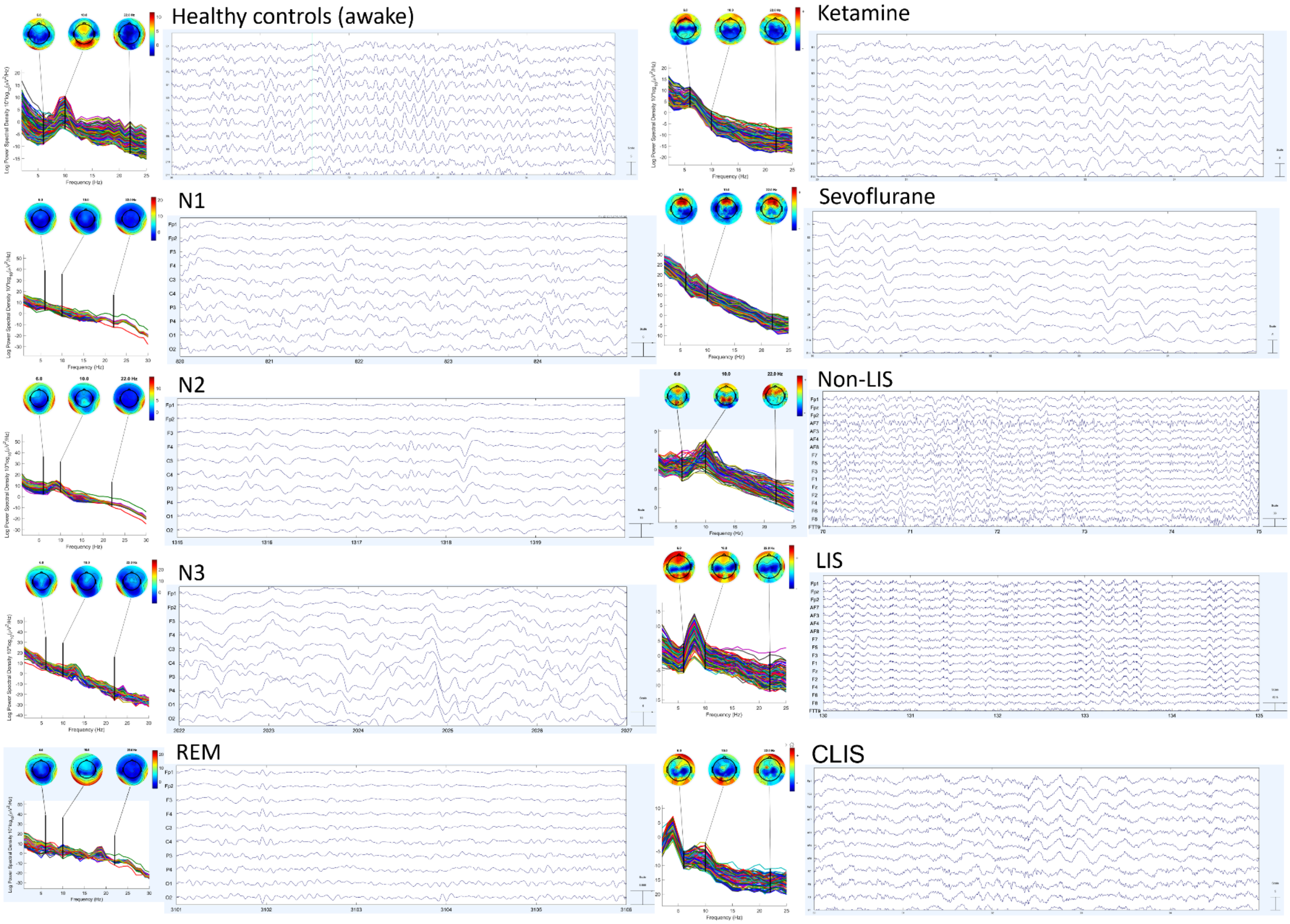

Supplementary Fig. 5: Sample of raw EEG data from each group, with the Power Spectral Density of the given EEG session

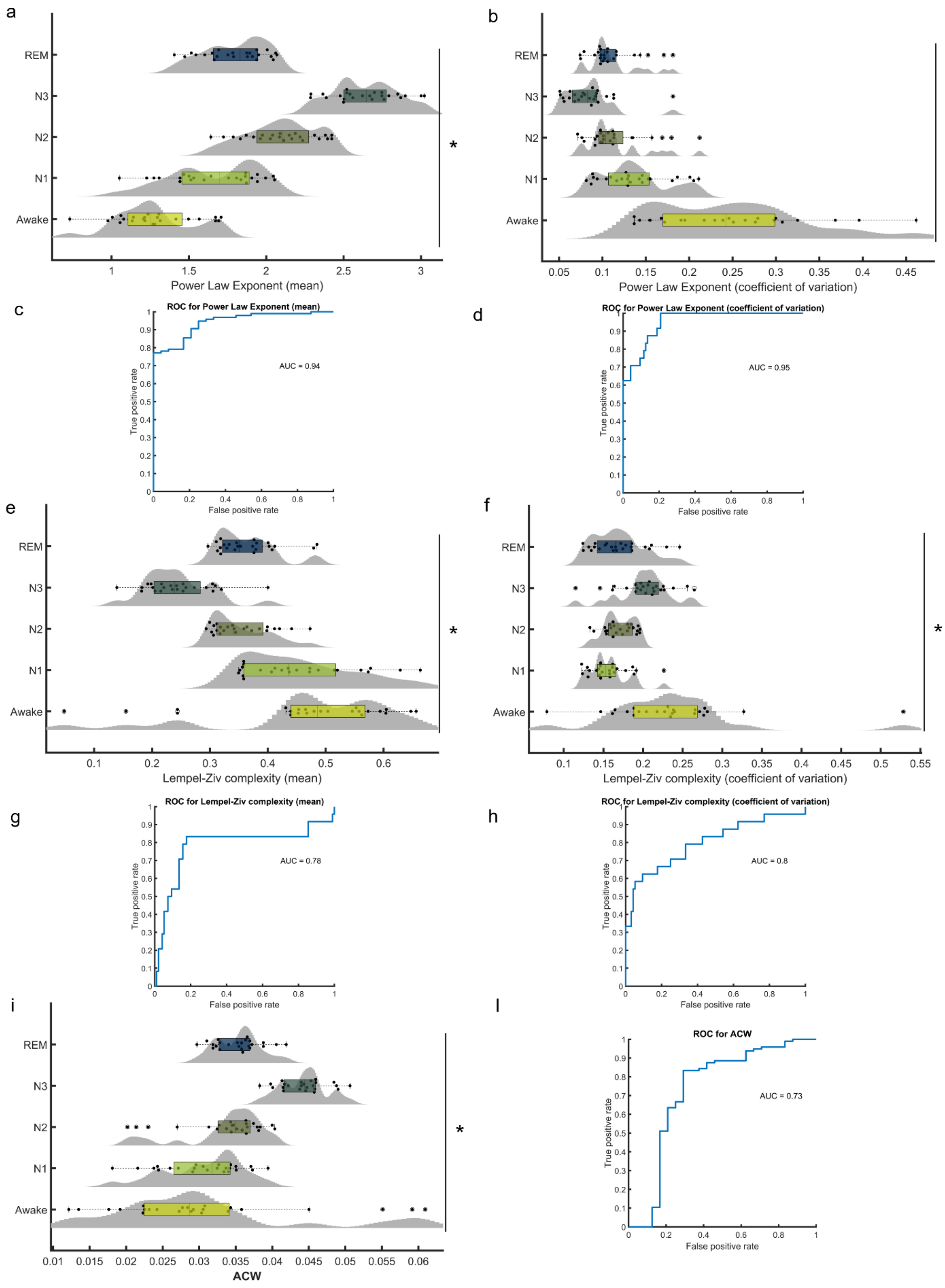

Supplementary Fig. 6: Sleep dataset analysis with 5-sec window, 50% overlap, and Autocorrelation window calculation.

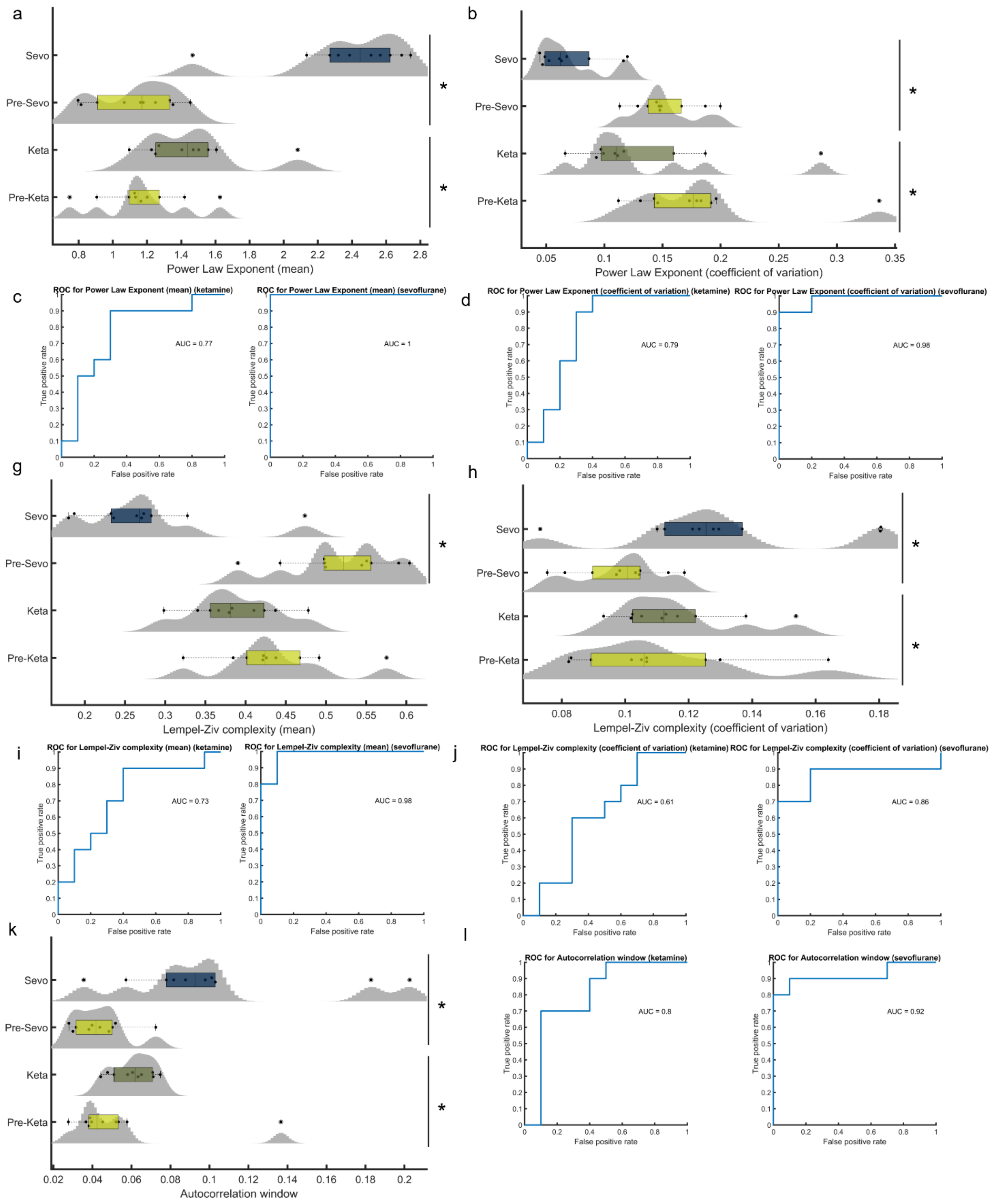

Supplementary Fig. 7: Analysis of the anesthesia dataset with a 5-sec window, 50% overlap, and Autocorrelation window calculation.

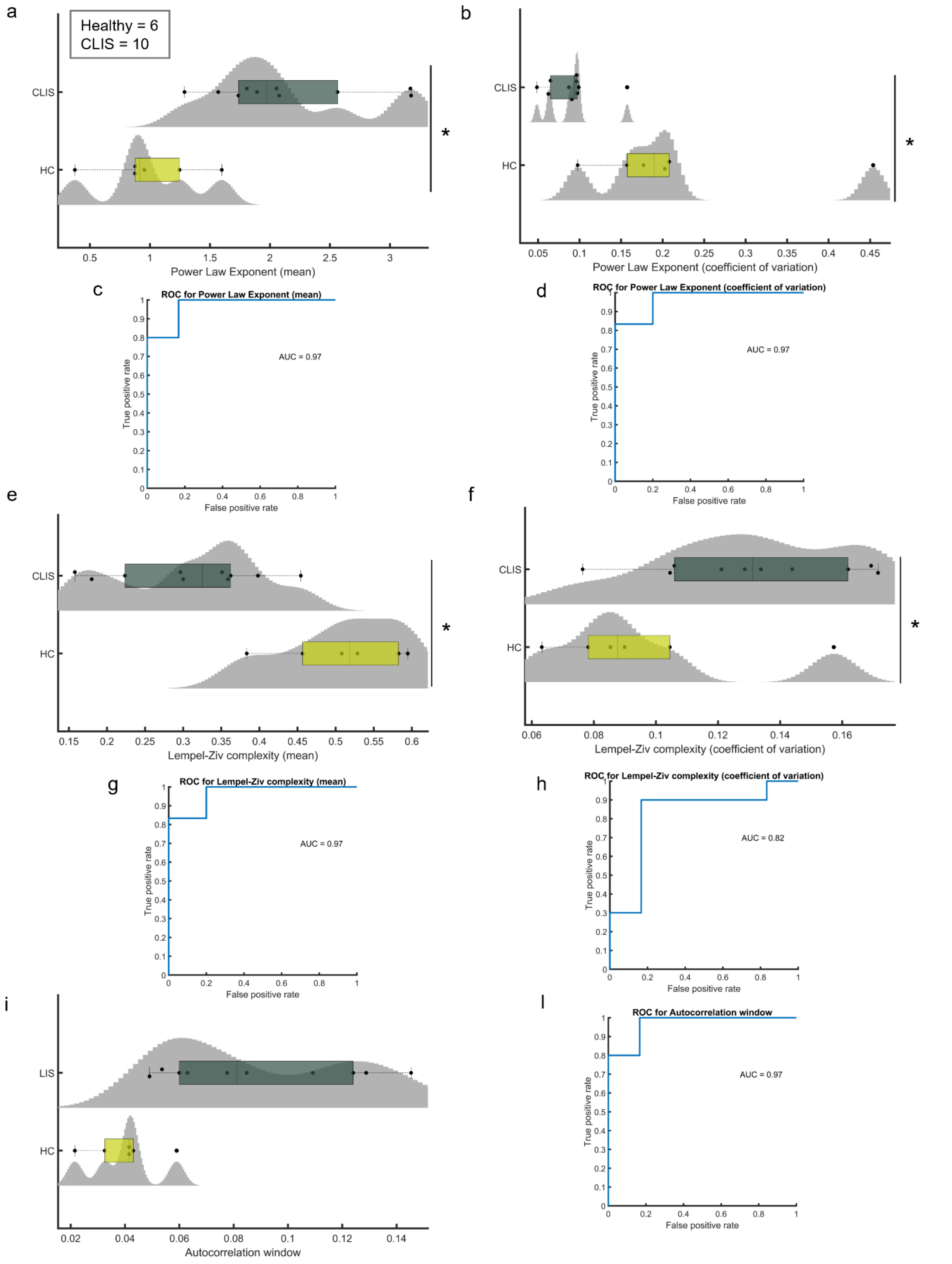

Supplementary Fig. 8: CLIS dataset analysis with 5-sec window, 50% overlap, and Autocorrelation window calculation.

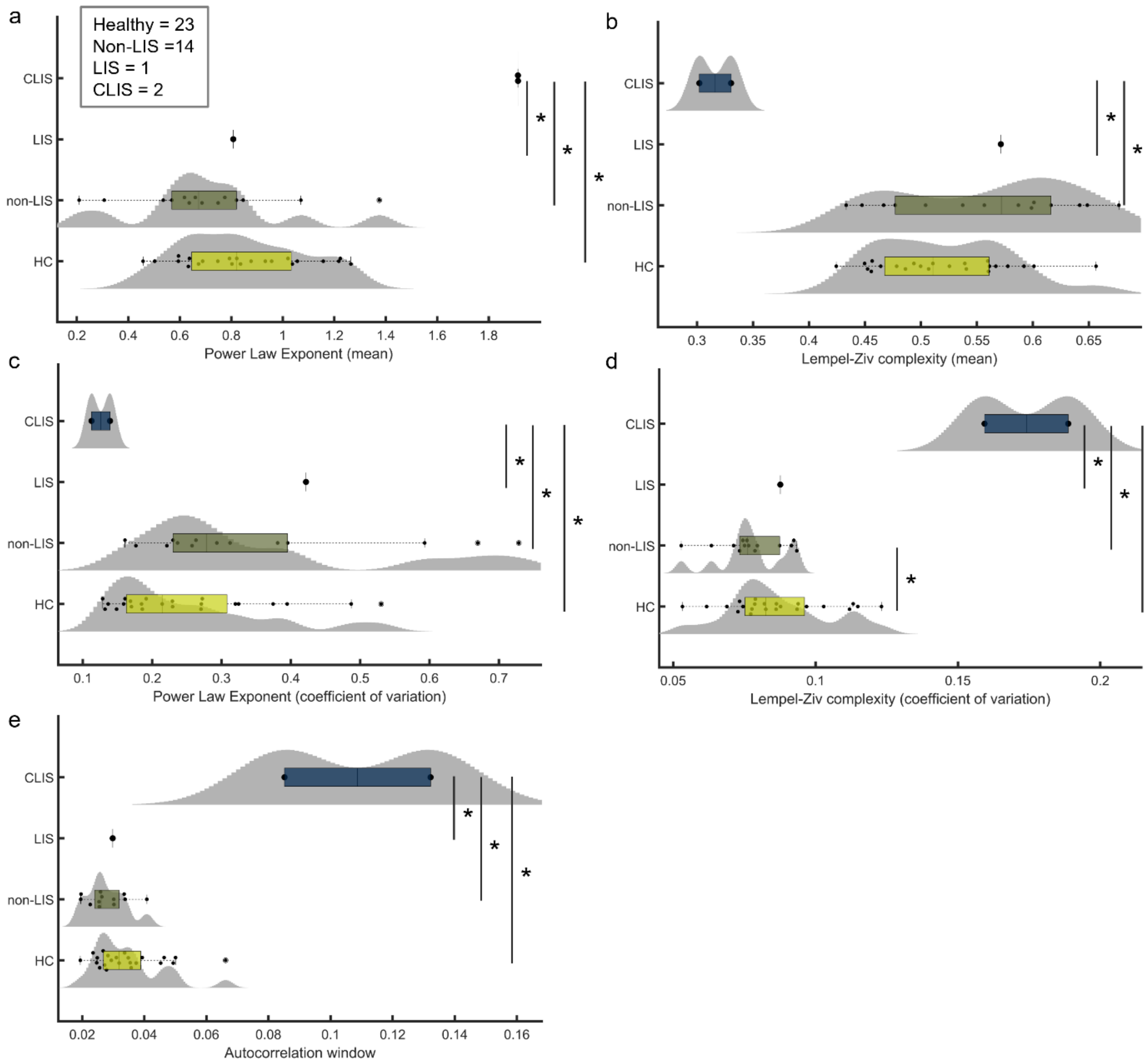

Supplementary Fig. 9: ALS dataset analysis with 5-sec window, 50% overlap, and Autocorrelation window calculation.

Supplementary Tables

Parameter	Awake state	Anaesthetic state	P-value
Sevoflurane anaesthesia			
HR (beats/min)	69.5±7.6	67.8±7.5	0.37
SBP (mmHg)	125.2±13.7	101.2±23.7	<0.01
DBP (mmHg)	71.2±9.6	56.3±17.9	<0.01
RR（times/min）	13.2±1.6	10.7±1.0	0.01
SpO_2（%）	98.5±1.4	99.0±0.6	0.08
PaO_2 (mmHg)	105.5±17.4	451.5±155.8	<0.01
$PaCO_2$ (mmHg)	38.9±3.5	39.1±3.7	0.28
PH	7.43±0.05	7.42±0.01	0.55
Ketamine anaesthesia			
HR (beats/min)	75.2±13.0	86.3±14.0	0.02
SBP (mmHg)	137.5±18.3	151.5±18.0	<0.01
DBP (mmHg)	73.2±15.2	84.9±8.3	0.01
RR（times/min）	13.4±1.6	11.4±1.3	0.04
SpO_2（%）	98.1±1.4	99.2±0.6	0.08
PaO_2 (mmHg)	105.5±12.1	451.5±150.8	<0.01
$PaCO_2$ (mmHg)	39.6±3.1	41.8±4.3	0.38
PH	7.43±0.05	7.42±0.01	0.75

Note: HR=heart rates; SBP=systolic blood pressure; DBP=diastolic blood pressure; RR=respiratory rates; SpO2=pulse oxygen saturation; PaO2 = partial oxygen pressure and PaCO2 = partial carbon dioxide pressure.

Supplementary Table 1: Clinical data before and after anaesthesia in two anaesthesia groups.

Supporting References

1. Honey, C. J. *et al.* Slow Cortical Dynamics and the Accumulation of Information over Long Timescales. *Neuron* **76**, 423–434 (2012).

2. Wolff, A. *et al.* The temporal signature of self: Temporal measures of resting-state EEG predict self-consciousness. *Hum Brain Mapp* (2019) doi:10.1002/hbm.24412.
