# Supplementary material for: Altered brain dynamics index levels of arousal in complete locked-in syndrome
Source: Commun Biol. 2023 Jul 20;6:757. doi: 10.1038/s42003-023-05109-1 (PMC10359418; doi:10.1038/s42003-023-05109-1)
Supplement: Supplementary file 2 — Description of Additional Supplementary Files [file 42003_2023_5109_MOESM2_ESM.pdf]

Description of Additional Supplementary Files

File name: Supplementary Data 1
Description: Numerical source data used to generate the plots in the figures. Each excel sheet corresponds to the data used for a plot of the figures (e.g., 2a, 2b, 2c, etc.).
