# Supplementary material for: Altered brain dynamics index levels of arousal in complete locked-in syndrome
Source: Commun Biol. 2023 Jul 20;6:757. doi: 10.1038/s42003-023-05109-1 (PMC10359418; doi:10.1038/s42003-023-05109-1)
Supplement: Supplementary file 4 — Reporting Summary [file 42003_2023_5109_MOESM4_ESM.pdf]

nature portfolio

Corresponding author(s): Federico Zilio

Last updated by author(s): Jun 30, 2023

Reporting Summary

Nature Portfolio wishes to improve the reproducibility of the work that we publish. This form provides structure for consistency and transparency in reporting. For further information on Nature Portfolio policies, see our Editorial Policies and the Editorial Policy Checklist.

Statistics

For all statistical analyses, confirm that the following items are present in the figure legend, table legend, main text, or Methods section.

n/a	Confirmed	
☐	☒	The exact sample size (*n*) for each experimental group/condition, given as a discrete number and unit of measurement
☐	☒	A statement on whether measurements were taken from distinct samples or whether the same sample was measured repeatedly
☐	☒	The statistical test(s) used AND whether they are one- or two-sided<br>*Only common tests should be described solely by name; describe more complex techniques in the Methods section.*
☐	☒	A description of all covariates tested
☐	☒	A description of any assumptions or corrections, such as tests of normality and adjustment for multiple comparisons
☐	☒	A full description of the statistical parameters including central tendency (e.g. means) or other basic estimates (e.g. regression coefficient) AND variation (e.g. standard deviation) or associated estimates of uncertainty (e.g. confidence intervals)
☐	☒	For null hypothesis testing, the test statistic (e.g. *F*, *t*, *r*) with confidence intervals, effect sizes, degrees of freedom and *P* value noted<br>*Give P values as exact values whenever suitable.*
☒	☐	For Bayesian analysis, information on the choice of priors and Markov chain Monte Carlo settings
☒	☐	For hierarchical and complex designs, identification of the appropriate level for tests and full reporting of outcomes
☒	☐	Estimates of effect sizes (e.g. Cohen's *d*, Pearson's *r*), indicating how they were calculated

Our web collection on statistics for biologists contains articles on many of the points above.

Software and code

Policy information about availability of computer code

Data collection	We re-used already collected data from previous EEG studies.
Data analysis	EEG preprocessing and the computations of power-law exponent and Lempel-Ziv complexity were performed in Matlab (The MathWorks, 2017b) and with the EEGLAB toolbox. Statistical analysis was performed with the Matlab 'Statistics and Machine Learning' Toolbox (version 2017b). Figures were created in Matlab (2017b). Code availability statement: "The Matlab codes are available on the following URL: https://github.com/Temporo-spatial/PLE_LZC"

For manuscripts utilizing custom algorithms or software that are central to the research but not yet described in published literature, software must be made available to editors and reviewers. We strongly encourage code deposition in a community repository (e.g. GitHub). See the Nature Portfolio guidelines for submitting code & software for further information.

Data

Policy information about availability of data

All manuscripts must include a data availability statement. This statement should provide the following information, where applicable:

- Accession codes, unique identifiers, or web links for publicly available datasets
- A description of any restrictions on data availability
- For clinical datasets or third party data, please ensure that the statement adheres to our policy

"The data that support the findings of this study are available on request from the corresponding author, upon reasonable request. The data are not publicly available due to privacy reasons."

Human research participants

Policy information about studies involving human research participants and Sex and Gender in Research.

Reporting on sex and gender	Neither the terms sex nor gender were used in our EEG analysis. The EEG analysis used the terms “subjects”, “participants”, and "patients" only.
Population characteristics	Sleep dataset: 23 healthy adults (age= 25.95 ± 6.53 years, male/female: 8/15) were included. Ketamine dataset: 10 right-handed surgical subjects (age=32.90 ± 9.48 years, male/female: 6/4) were included. Sevoflurane dataset: 10 right-handed surgical subjects (age = 41.4 ± 13.1 years, male/female: 8/2) were included. CLIS with ALS dataset: 10 subjects with complete locked-in syndrome - CLIS (age= 47.1 ± 20.74 years, male/female: 5/5). ALS dataset: 14 non-locked-in Syndrome ALS patients (age=58.5 ± 11.78 years, male/female: 9/1, 4 n.a.) with ALSFRS-R scores of 3 to 40 (min = 0, max = 48), a single female ALS patient (age = 52 years) in locked-in syndrome (ALSFRS-R = 1), and 2 ALS patients (male/woman: 1/1, age: 43/64) in CLIS (ALSFRS-R = 0).
Recruitment	*Describe how participants were recruited. Outline any potential self-selection bias or other biases that may be present and how these are likely to impact results.*
Ethics oversight	All participants (or their legal guardians) gave their informed written consent before participating. This research was approved by the respective Universities/Hospitals depending on the origin of the dataset (sleep dataset: Western University Health Science Research Ethics Board; Anesthesia dataset: Huashan Hospital, Fudan University; CLIS dataset: Medical Faculty of the University of Tübingen; ALS dataset: Max Planck Society Ethics Committee). This study was carried out in accordance with the Declaration of Helsinki guidelines.

Note that full information on the approval of the study protocol must also be provided in the manuscript.

Field-specific reporting

Please select the one below that is the best fit for your research. If you are not sure, read the appropriate sections before making your selection.

☒ Life sciences ☐ Behavioural & social sciences ☐ Ecological, evolutionary & environmental sciences

For a reference copy of the document with all sections, see nature.com/documents/nr-reporting-summary-flat.pdf

Life sciences study design

All studies must disclose on these points even when the disclosure is negative.

Sample size	For the EEG analyses we re-used data from previous studies. Sleep dataset: 23 healthy adults (age= 25.95 ± 6.53 years, male/female: 8/15) were included. Ketamine dataset: 10 right-handed surgical subjects (age=32.90 ± 9.48 years, male/female: 6/4) were included. Sevoflurane dataset: 10 right-handed surgical subjects (age = 41.4 ± 13.1 years, male/female: 8/2) were included. CLIS with ALS dataset: 10 subjects with complete locked-in syndrome - CLIS (age= 47.1 ± 20.74 years, male/female: 5/5). ALS dataset: 14 non-locked-in Syndrome ALS patients (age=58.5 ± 11.78 years, male/female: 9/1, 4 n.a.) with ALSFRS-R scores of 3 to 40 (min = 0, max = 48), a single female ALS patient (age = 52 years) in locked-in syndrome (ALSFRS-R = 1), and 2 ALS patients (male/woman: 1/1, age: 43/64) in CLIS (ALSFRS-R = 0).
Data exclusions	No data or subjects were excluded from the analysis after pre-processing.
Replication	We used four different datasets with different conditions to verify, compare, and replicate our findings. We used subjects with different states of consciousness due to sleep state, or pharmacological induced states (anesthesia) to validate our measurements (PLE and LZC) as benchmarks of a wide range of states of consciousness (awake, sleep stages N1-2-3-REM, ketamine, sevoflurane). Then we replicated our findings in the two data sets composed of participants with complete locked-in syndrome (and healthy controls) and amyotrophic lateral sclerosis (with different degrees of severity + healthy controls).
Randomization	In the case of the sleep and anesthesia datasets, we use a longitudinal study design, so that we compare the same subjects across different

Randomization	states: awake, N1, N2, N3 and REM in the case of sleep dataset; awake vs ketamine and awake vs sevoflurane in the case of the anesthesia dataset.
Blinding	Our study followed an observational retrospective design, so the blinding process was not relevant for the analysis.

Reporting for specific materials, systems and methods

We require information from authors about some types of materials, experimental systems and methods used in many studies. Here, indicate whether each material, system or method listed is relevant to your study. If you are not sure if a list item applies to your research, read the appropriate section before selecting a response.

Materials & experimental systems

n/a	Involved in the study	
☒	☐	Antibodies
☒	☐	Eukaryotic cell lines
☒	☐	Palaeontology and archaeology
☒	☐	Animals and other organisms
☒	☐	Clinical data
☒	☐	Dual use research of concern

Methods

n/a	Involved in the study	
☒	☐	ChIP-seq
☒	☐	Flow cytometry
☒	☐	MRI-based neuroimaging
